# Evaluation of respondent-driven sampling in seven studies of people who use drugs from rural populations: findings from the Rural Opioid Initiative

**DOI:** 10.1186/s12874-024-02206-5

**Published:** 2024-04-23

**Authors:** Abby E. Rudolph, Robin M. Nance, Georgiy Bobashev, Daniel Brook, Wajiha Akhtar, Ryan Cook, Hannah L. Cooper, Peter D. Friedmann, Simon D. W. Frost, Vivian F. Go, Wiley D. Jenkins, Philip T. Korthuis, William C. Miller, Mai T. Pho, Stephanie A. Ruderman, David W. Seal, Thomas J. Stopka, Ryan P. Westergaard, April M. Young, William A. Zule, Judith I. Tsui, Heidi M. Crane, Bridget M. Whitney, Joseph A. C. Delaney

**Affiliations:** 1 Department of Epidemiology and Biostatistics, Temple University College of Public Health, 1301 Cecil B Moore Avenue, Ritter Annex 905, Philadelphia, PA USA; 2 Harborview Medical Center, University of Washington, 325 9th Ave, Box 359931, Seattle, WA 98106 USA; 3 RTI International, 3040 East Cornwallis Road, P.O. Box 12194, Research Triangle Park, NC 27709-2194 USA; 4 Division of Epidemiology, College of Public Health, Ohio State University, Columbus, Ohio 43210 USA; 5 University of Wisconsin-Madison, Population Health Institute, 610 Walnut Street, 575 WARF, Madison, WI 53726 USA; 6 General Internal Medicine and Geriatrics, Oregon Health & Science University, 3181 SW Sam Jackson Park Road, Portland, OR 97239-3098 USA; 7 Rollins School of Public Health, Emory University, Grace Crum Rollins Building, 1518 Clifton Road, Atlanta, Georgia 30322 USA; 8 Baystate Medical Center—University of Massachusetts, Office of Research, UMass Chan Medical School – Baystate, 3601 Main Street, 3rd Floor, Springfield, MA 01199 USA; 9 Microsoft Premonition, Microsoft Building 99, 14820 NE 36th St. Redmond, Seattle, WA 98052 USA; 10 Department of Infectious Disease Epidemiology, London School of Hygiene and Tropical Medicine, Keppel Street, London, WC1E 7HT UK; 11 University of North Carolina—Chapel Hill, 363 Rosenau Hall, CB# 7440, Chapel Hill, NC 27599 USA; 12 Southern Illinois University, 201 E Madison Street, Springfield, IL 62702 USA; 13 Oregon Health & Science University, 3270 Southwest Pavilion Loop OHSU Physicians Pavilion, Suite 350, Portland, OR 97239 USA; 14 The Ohio State University, 302 Cunz Hall, 1841 Neil Ave, Columbus, OH 43210 USA; 15 University of Chicago, 5841 S. Maryland Avenue, Chicago, IL 60637 USA; 16 Tulane University, 1440 Canal Street, Suite 2210, New Orleans, LA 70112 USA; 17 Tufts University School of Medicine, Public Health and Community Medicine, 136 Harrison Avenue, Boston, MA 02111 USA; 18 University of Wisconsin-Madison, 1685 Highland Avenue, 5th Floor, Madison, WI 53705-2281 USA; 19 University of Kentucky, 760 Press Avenue, Suite 280, Lexington, KY 40536 USA; 20 Harborview Medical Center, University of Washington and University of Manitoba, University of Washington, 325 9th Ave, Box 359931, Seattle, WA 98106 USA

**Keywords:** Respondent-driven sampling, Opioids, Methamphetamine, Drug use, Rural health

## Abstract

**Background:**

Accurate prevalence estimates of drug use and its harms are important to characterize burden and develop interventions to reduce negative health outcomes and disparities. Lack of a sampling frame for marginalized/stigmatized populations, including persons who use drugs (PWUD) in rural settings, makes this challenging. Respondent-driven sampling (RDS) is frequently used to recruit PWUD. However, the validity of RDS-generated population-level prevalence estimates relies on assumptions that should be evaluated.

**Methods:**

RDS was used to recruit PWUD across seven Rural Opioid Initiative studies between 2018-2020. To evaluate RDS assumptions, we computed recruitment homophily and design effects, generated convergence and bottleneck plots, and tested for recruitment and degree differences. We compared sample proportions with three RDS-adjusted estimators (two variations of RDS-I and RDS-II) for five variables of interest (past 30-day use of heroin, fentanyl, and methamphetamine; past 6-month homelessness; and being positive for hepatitis C virus (HCV) antibody) using linear regression with robust confidence intervals. We compared regression estimates for the associations between HCV positive antibody status and (a) heroin use, (b) fentanyl use, and (c) age using RDS-1 and RDS-II probability weights and no weights using logistic and modified Poisson regression and random-effects meta-analyses.

**Results:**

Among 2,842 PWUD, median age was 34 years and 43% were female. Most participants (54%) reported opioids as their drug of choice, however regional differences were present (e.g., methamphetamine range: 4-52%). Many recruitment chains were not long enough to achieve sample equilibrium. Recruitment homophily was present for some variables. Differences with respect to recruitment and degree varied across studies. Prevalence estimates varied only slightly with different RDS weighting approaches, most confidence intervals overlapped. Variations in measures of association varied little based on weighting approach.

**Conclusions:**

RDS was a useful recruitment tool for PWUD in rural settings. However, several violations of key RDS assumptions were observed which slightly impacts estimation of proportion although not associations.

**Supplementary Information:**

The online version contains supplementary material available at 10.1186/s12874-024-02206-5.

## Background

The United States (US) opioid overdose epidemic remains a challenging public health issue [1–4], particularly as the epidemic evolves and becomes more complex, with the increasing co-use of stimulants and opioids [5]. The absence of a sampling frame for marginalized and/or highly stigmatized populations (e.g., people who use drugs [PWUD]) makes it challenging to generate accurate prevalence and incidence estimates of drug-related risk behaviors and health outcomes, especially in rural settings. Respondent-driven sampling (RDS), a modified form of chain referral sampling, has successfully been used to recruit PWUD in a range of research contexts. While there are examples of using RDS to recruit PWUD in rural settings [6, 7], this has been more limited. RDS uses incentives for both study participation and peer recruitment and uses sampling weights to offset non-random recruitment. The validity of the RDS-adjusted prevalence estimates, however, relies on assumptions which are often not empirically evaluated or reported in the published literature [8] and have been violated in some research with rural PWUD [6, 7]. In particular, the presence and impact of homophily, or the tendency for participants to preferentially recruit peers with similar behaviors or characteristics, should be assessed. Further, different approaches and RDS estimators have been developed to offset sampling biases and each has different assumptions.

### RDS-I estimator

The RDS-I estimator, also known as the Salganik-Heckathorn estimator, is based largely on Markov chain theory and social network theory. RDS-I sampling weights incorporate information on cross-group recruitment and personal network size/degree (hereinafter referred to as degree), defined as the number of people in the target population that a respondent reports knowing [9, 10]. Briefly, the RDS-I estimator assumes that: 1. Respondents are members of a target population, which is completely connected and where every member of the population can reach every other member through their connections to others in the population; 2. Respondents can accurately report their degree; 3. Individuals recruit from their personal network at random and recruitment ties are reciprocal, such that one’s likelihood of being recruited to the study is proportional to degree; 4. Recruitment patterns depend only on the recruiter and not on the recruiter’s recruiter; 5. Cross-group recruitment is sufficient (i.e., recruitment homophily is low), such that once a respondent with a specific characteristic is recruited, future recruits are not exclusively those with the same characteristic; and 6. Recruitment is sufficiently deep to overcome bias introduced by the convenience sample of seeds [11].

### RDS-II estimator

The RDS-II estimator, also known as the Volz-Heckathorn estimator, was proposed to address some of the biases associated with the RDS-I estimator. Like the RDS-I estimator, weights are based on degree, however unlike the RDS-I estimator, weights are based on each individual’s degree instead of the average degree in a group. Additionally, it does not account for homophily, or cross-group recruitment. The RDS-II estimator assumes that: 1. Respondents are members of a completely connected network with a finite (but large) population size; 2. Recruitment ties are reciprocal; 3. Respondents can accurately report their degree; 4. Respondents recruit those from their personal network at random; 5. Each respondent recruits only one peer and sampling occurs with replacement; and 6. The sampling fraction is small but recruitment is sufficiently deep to overcome bias introduced by the convenience sample of seeds [12, 13].

Both RDS estimators make complex assumptions that are often difficult to fully evaluate using empirical data [13] and there is not a clear test of which estimator is less biased [14]. A previous RDS analysis comparing chain length suggested that studies with a lot of seeds and short chains might converge more quickly on the underlying population levels using the RDS-II estimator [14]. However, the estimates of proportion would still be susceptible to high-degree participants being captured differentially. Nevertheless, cases of extreme homophily or homophily among the seeds (themselves) could impact findings, particularly in a rural environment with a large number of seeds, and it is not possible to tell which estimator actually has the least bias using empirical data. Simulation studies [15], using assumptions about the data generating process, often arrive at different conclusions than empirical work, highlighting the need to better understand the recruitment process using empirical data.

Prior studies have suggested that RDS studies need to more comprehensively report the methods used and consider following the STROBE-RDS guidelines to make approaches and assumptions clearer [16, 17]. In this study we aimed to: 1. Describe the RDS methodology used in a multi-site consortium of PWUD in rural settings; 2. Evaluate whether the RDS assumptions were met in the seven ROI studies; 3. Compare sample prevalence estimates and RDS-adjusted prevalence estimates; and 4. Compare the direction and strength of associations observed when regression analyses are conducted using unweighted, RDS-I and RDS-II-weighted data.

## Methods

### Consortium

The National Institute on Drug Abuse (NIDA), in partnership with the Appalachian Regional Commission (ARC), the Centers for Disease Control and Prevention (CDC), and the Substance Abuse and Mental Health Services Administration (SAMHSA), funded the Rural Opioid Initiative (ROI) cooperative agreement consortium to better understand and address the opioid and injection drug use crisis across rural America [18].

### Recruitment and eligibility

The ROI consortium consists of 8 studies, 7 of which had relevant data available and are included here. Each ROI study was located in a rural community impacted by the opioid overdose epidemic (see Supplemental Figure 1 [ROI Map]; Illinois: IL, Kentucky: KY, North Carolina: NC, New England: NE [11 rural counties in Massachusetts, New Hampshire, and Vermont], Ohio: OH, Oregon: OR, Wisconsin: WI, and West Virginia: WV) [18]. Participants were recruited using RDS between 2018-2020. Given regional differences in demographic characteristics and substance use patterns, there were slight differences in the eligibility criteria, approach for identifying seeds, incentives, number of peer recruits permitted per person, and the wording of the degree question (see details for each study in Supplemental Table 1 [STROBE Checklist]). In brief, participants were eligible to participate if they were residents of the study area, met age requirements (≥18 years of age for 5 sites and ≥15 years of age for two sites) and reported injection of any drug to get high or non-injection use of opioids to get high in the past 30 days.

**Fig. 1:**
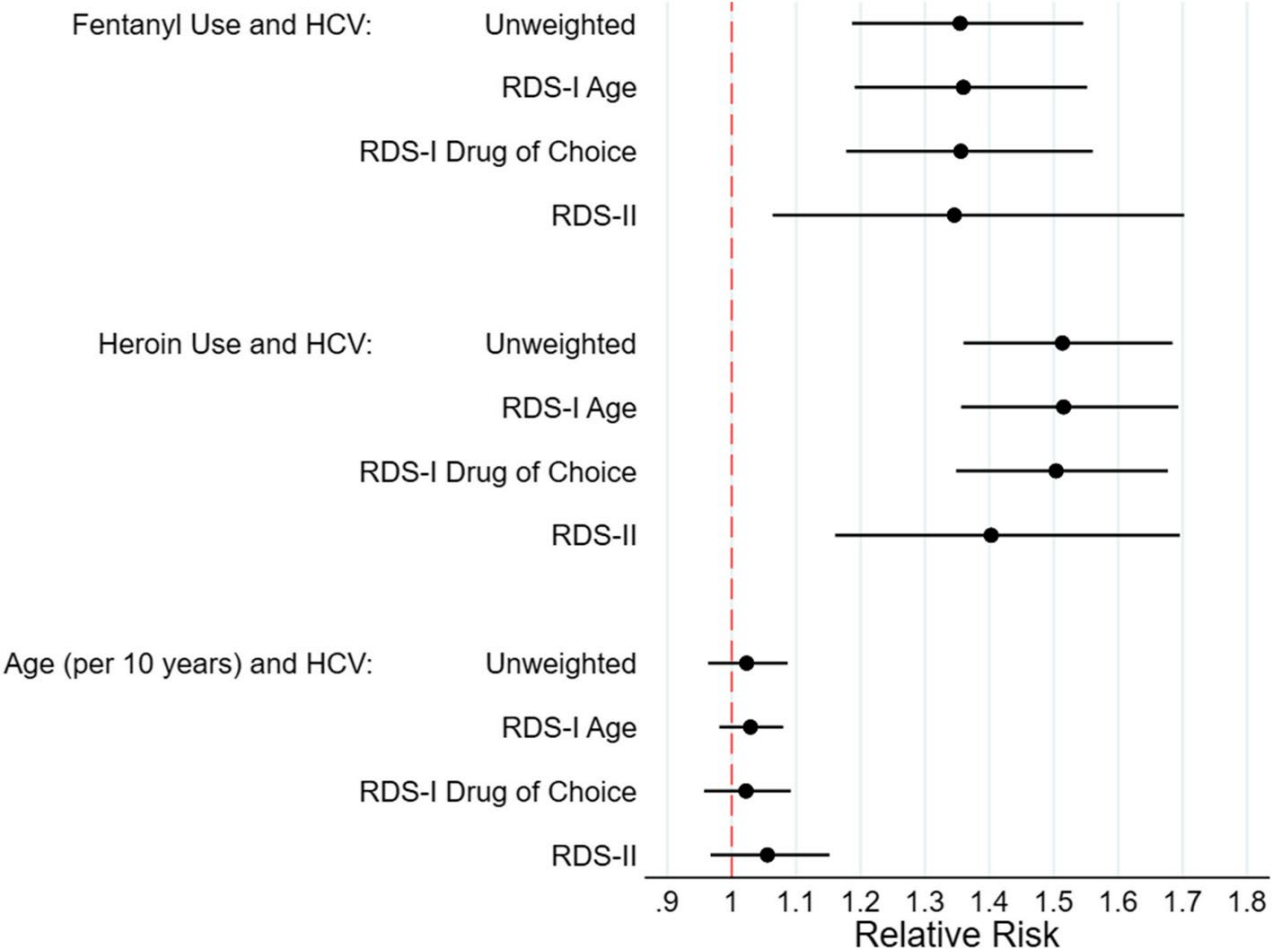
Forest plots of unweighted and RDS-weighted measures of association for the relationship between (**a**) fentanyl use, (**b**) heroin use, and (**c**) age and positive Hepatitis C Virus antibody status. Abbreviations: HCV, hepatitis C virus. Reference period for fentanyl and heroin use: past 30 days

Recruitment was initiated by seeds. In KY, PWUD with large networks identified in a previous study [19] were selected as seeds, but other sites did not impose network size criteria (Table 1). Across all seven sites, eligible and enrolled peer-recruiters and seeds could recruit 3-7 eligible peers, with this process continuing until sample size goals were met. Incentives were offered for initial participation ($20-$45) and peer-referral ($10-$20 per eligible peer referred/enrolled), depending on study and local research conditions. Each study collected quantitative data from PWUD using a harmonized instrument. A standard degree question assessed each participant’s self-reported degree (see wording in Supplemental Table 1) at all sites except NE, where degree was estimated by counting the number of network members listed in the social network inventory who were perceived to inject drugs or use opioids.

**Table 1:** Key characteristics of RDS recruitment methodology in the Rural Opioid Initiative by study

		**Study**						
	**Overall**	**IL**	**KY**	**NC**	**NE**	**OH**	**OR**	**WI**
**Personal network size used as a seed selection criterion**	1/7	No	Yes^a^	No	No	No	No	No
**Total number of seeds**	562	53	48	50	51	45	42	273
Non-generative seeds	251 (45%)	38 (72%)	24 (50%)	13 (26%)	13 (25%)	23 (51%)	18 (43%)	122 (45%)
Seeds with ≥5 waves	46 (8%)	1 (2%)	4 (8%)	6 (12%)	11 (22%)	3 (7%)	4 (10%)	17 (6%)
Wave size range, median (range)	1 (0-14)	0 (0-5)	0.5 (0-13)	1 (0-11)	1 (0-14)	0 (0-13)	1 (0-7)	1 (0-11)
**Coupons per recruiter**^b^	3-7	6	3	3-4	3-7	4-5	3-4	3-5
**Incentive for participation**^c^	$20-40	$40 for completing the survey	$45 for completing the survey and rapid testing	$40 for completing the survey	$20 for completing the survey and rapid testing	$25 for completing the survey	$25 for completing the survey	$20 for completing the survey and rapid testing
**Incentive for recruitment**	$10-20	$20 per eligible referral	$10 per eligible referral who enrolled	$20 per eligible referral who enrolled	$10 per eligible referral who completed all study activities	$10 per eligible referral	$10 per eligible referral	$10 per eligible referral who enrolled
**Duplicate participants identified**	20	2	3	0	0	3	1	14
Downstream recruits of duplicates	31	7	0	0	0	6	0	18

*Abbreviations*: *IL* Illinois, *KY* Kentucky, *NC* North Carolina, *NE* New England (Massachusetts, New Hampshire, Vermont), *OH* Ohio, *OR* Oregon, *WI* Wisconsin

^a^Seeds had to be “highly connected,” which was defined as reporting having used drugs with ≥10 people in the past 30 days for women and ≥20 people in the past 30 days for men; thresholds determined using the top quartile of network size based on gender-stratified analyses of preliminary data from an online survey

^b^Range given for sites that changed coupon policies while the study was ongoing

^c^For completing study activities (baseline survey and biological testing)

### RDS diagnostics

Cytoscape software was used to visualize recruitment chains across studies (see Supplemental Figure 2 [recruitment diagrams]) [20, 21]. We generated convergence and bottleneck plots and computed degree and recruitment differences, recruitment homophily, and design effect for key, self-reported variables collected using the core ROI baseline questionnaire (age [continuous and categorical, <25, 25-34, 35-44, 45-54, and ≥55 years], current drug of choice for ‘getting high’ [heroin, methamphetamine, or other drug], past 30-day use of heroin, past 30-day use of fentanyl, past 30-day use of methamphetamine, being positive for HCV antibody, and homelessness in the past 6 months).

Differences in degree are presented as degree ratios relative to a reference group (i.e., mean network size for those with a particular attribute/characteristic category compared to those with a different value), and recruitment differences are similarly presented [20, 21]. We tested for degree and recruitment differences using the *RDS package* (version 0.9-3) [22] and *chords package* (version 0.95.4) in R. Recruitment homophily was measured as the ratio of the number of recruits who have the same attribute of interest as their recruiter to the number we would expect by chance for that recruitment chain. When recruitment homophily for a particular variable is close to 1.0, it indicates that there is not much recruitment homophily on that variable (so smaller sample sizes will be needed) [23]. When homophily is greater than 1, individuals tend to recruit peers who are similar with respect to that attribute; homophily values of ≥1.3 indicate high homophily. A homophily value <1 indicates that individuals tend to recruit peers who are dissimilar with respect to the characteristic/attribute of interest. Design effect is measured as the ratio of the observed variance under RDS to that which would be expected for the same estimate under a simple random sampling scheme and indicates the increase in sample size required when using RDS to achieve the same power versus simple random sampling. Lastly, we used the model-based approach of Berchenko et al. [24] to incorporate information on the timing of recruitment to estimate how much faster high-degree participants were recruited using the theta parameter, also known as the coefficient of discoverability. We computed the average degree and number of peer recruits by each variable of interest.

### Prevalence estimates

We computed sample proportions, or unweighted prevalence estimates, and three RDS-adjusted population proportions, or population prevalence estimates, for five key variables of interest: past 30-day use of heroin, fentanyl, and methamphetamine; homelessness in the past 6 months; and HCV positive antibody status. The RDS-adjusted population proportions were calculated using: [1] RDS-I accounting for homophily on drug of choice; [2] RDS-I accounting for homophily on age (categorical), and [3] RDS-II. Seeds were included in analyses and participants with missing data on key variables of interest were removed. All prevalence estimates were computed using linear regression with robust confidence intervals. Sensitivity analyses were conducted to compare the tree bootstrap approach of Baraff et al. to the use of robust confidence intervals [25]. We explored the possibility of seed bias by removing seeds and comparing estimates, similar to approaches used by Lachowsky et al. [26] who explored various seed deletion approaches. We also compared the magnitude and direction of effect estimates for the associations between the past 30-day fentanyl use and HCV positive antibody status, past 30-day heroin use and HCV positive antibody status, and age and HCV positive antibody status when RDS sampling weights were and were not applied to the data using relative risk regression using the modified Poisson regression approach of Zou et al. [27] and traditional logistic regression. To get a pooled estimate across studies we used an inverse-variance weighted meta-analytic approach to pool estimates [28]. Unless otherwise noted, regression analyses were completed in Stata version 17 (StataCorp, College Station, TX).

## Results

A total of 2,893 PWUD from 7 ROI studies were enrolled. After removing 20 duplicate participants and their 31 downstream recruits (Table 1; see details for each study in Supplemental Table 1 [STROBE Checklist]), the resulting overall sample size was 2,842 (sample size range: 166-973 PWUD per study, Table 2). The number of seeds per study ranged from 42-53 for all studies other than WI, the largest study, which used 273 seeds (Table 1). Non-generative seeds were common, with 4 of 7 studies having 43-51% of seeds who did not recruit any additional participants (range 25-72%, Table 1). The proportion of seeds with ≥5 waves of recruitment was low across studies (overall: 8%, range: 2-22%), with an overall median wave size of 1 (range: 0-14); no study had a median wave size >1 (see Supplemental Figure 2 [recruitment diagrams]).

**Table 2:** Characteristics of participants and substance use patterns in the Rural Opioid Initiative by study

		**Study**						
	**Overall**	**IL**	**KY**	**NC**	**NE**	**OH**	**OR**	**WI**
**N**	2,842	166	338	350	589	252	174	973
**Age**, median (IQR)	34 (28-42)	40 (31-47)	35 (29-41)	32 (27-42)	34 (28-42)	38 (32-47)	36 (29-45)	33 (27-40)
**Female**	1,212 (43%)	70 (42%)	144 (43%)	168 (48%)	243 (41%)	127 (50%)	75 (43%)	385 (40%)
**Race/Ethnicity**								
White	2,346 (83%)	141 (85%)	330 (98%)	237 (68%)	523 (89%)	222 (88%)	135 (78%)	758 (78%)
Black or African American	80 (3%)	17 (10%)	2 (<1%)	5 (1%)	6 (1%)	13 (5%)	3 (2%)	34 (3%)
Native American	206 (7%)	3 (2%)	1 (<1%)	77 (22%)	9 (2%)	5 (2%)	9 (5%)	102 (10%)
Other/Unknown	96 (3%)	3 (2%)	4 (1%)	12 (3%)	23 (4%)	7 (3%)	11 (6%)	36 (4%)
Hispanic^a^	114 (4%)	2 (1%)	1 (<1%)	19 (5%)	28 (5%)	5 (2%)	16 (9%)	43 (4%)
**Experienced homelessness**^b^	1,519 (53%)	80 (48%)	123 (36%)	151 (43%)	332 (56%)	127 (50%)	119 (68%)	587 (60%)
**Preferred drug for getting high**								
Opioids^c^	1,533 (54%)	80 (48%)	206 (61%)	171 (49%)	452 (77%)	179 (71%)	78 (45%)	367 (38%)
*Heroin*^*^*^	*1,079 (38%)*	*33 (20%)*	*103 (30%)*	*106 (30%)*	*351 (60%)*	*120 (48%)*	*69 (40%)*	*297 (31%)*
*Street fentanyl/carfentanil*^*^*^	*62 (2%)*	*2 (1%)*	*1 (<1%)*	*11 (3%)*	*23 (4%)*	*21 (8%)*	*0*	*4 (<1%)*
*Prescription opioids*^*^*^	*255 (9%)*	*31 (19%)*	*63 (19%)*	*44 (13%)*	*44 (7%)*	*29 (12%)*	*8 (5%)*	*36 (4%)*
*Buprenorphine*^*^*^	*79 (3%)*	*5 (3%)*	*36 (11%)*	*4 (1%)*	*25 (4%)*	*8 (3%)*	*1 (1%)*	*0*
*Methadone*^*^*^	*40 (1%)*	*9 (5%)*	*3 (1%)*	*6 (2%)*	*7 (1%)*	*1 (<1%)*	*0*	*14 (1%)*
Methamphetamine	1,016 (36%)	68 (41%)	108 (32%)	158 (45%)	23 (4%)	59 (23%)	91 (52%)	509 (52%)
Cocaine/crack	163 (6%)	12 (7%)	7 (2%)	14 (4%)	95 (16%)	8 (3%)	1 (1%)	26 (3%)
Benzodiazepines	39 (1%)	4 (2%)	6 (2%)	5 (1%)	4 (1%)	1 (<1%)	1 (1%)	18 (2%)
Other	76 (3%)	2 (1%)	11 (3%)	2 (1%)	15 (3%)	5 (2%)	3 (2%)	38 (4%)
**Drugs used in past 30 days**								
Opioids^c^	2,437 (86%)	139 (84%)	299 (88%)	298 (85%)	587 (99%)	235 (93%)	133 (76%)	746 (77%)
*Heroin*^*^*^	*1,963 (69%)*	*78 (47%)*	*230 (68%)*	*230 (66%)*	*531 (90%)*	*187 (78%)*	*105 (60%)*	*592 (61%)*
*Street fentanyl/carfentanil*^*^*^	*1,020 (36%)*	*42 (25%)*	*95 (28%)*	*160 (46%)*	*370 (63%)*	*150 (60%)*	*19 (11%)*	*184 (19%)*
*Prescription opioids*^*^*^	*1,615 (57%)*	*113 (68%)*	*211 (62%)*	*224 (64%)*	*339 (58%)*	*129 (51%)*	*67 (39%)*	*532 (55%)*
*Buprenorphine*^*^*^	*1,124 (40%)*	*79 (48%)*	*197 (58%)*	*142 (41%)*	*304 (52%)*	*113 (45%)*	*18 (10%)*	*271 (28%)*
*Methadone*^*^*^	*621 (22%)*	*29 (17%)*	*51 (15%)*	*60 (17%)*	*171 (29%)*	*34 (13%)*	*27 (16%)*	*249 (26%)*
Methamphetamine	2,148 (76%)	132 (80%)	265 (78%)	325 (93%)	203 (34%)	199 (79%)	168 (97%)	856 (88%)
Cocaine/crack	1,224 (43%)	76 (46%)	74 (22%)	82 (23%)	451 (77%)	103 (41%)	14 (8%)	424 (44%)
Benzodiazepines	1,325 (47%)	102 (61%)	147 (43%)	177 (51%)	300 (51%)	122 (48%)	47 (27%)	430 (44%)
Other	985 (35%)	49 (30%)	165 (49%)	77 (22%)	270 (46%)	123 (49%)	26 (15%)	275 (28%)
Multiple classes of drugs used^d^	2,404 (85%)	142 (86%)	294 (87%)	299 (85%)	517 (88%)	224 (89%)	137 (79%)	791 (81%)
**Ever injected drugs**^e^	2,610 (92%)	137 (83%)	290 (86%)	330 (94%)	499 (85%)	220 (87%)	161 (93%)	973 (100%)
**Injection drug use in past 30 days**^e^	2,420 (85%)	121 (73%)	245 (72%)	299 (85%)	431 (73%)	200 (79%)	153 (88%)	971 (>99%)
**HCV positive antibody status**^f^	1,353 (52%)	66 (45%)	208 (62%)	144 (68%)	325 (59%)	170 (72%)	86 (54%)	354 (36%)

Data presented as n (%) unless otherwise indicated

*Abbreviations*: *IL* Illinois, *KY* Kentucky, *NC* North Carolina, *NE* New England (Massachusetts, New Hampshire, Vermont), *OH* Ohio, *OR* Oregon, *WI* Wisconsin

^a^Race/ethnicity are mutually exclusive categories. Hispanic includes everyone who is Hispanic. White and Black race include those who are White or Black and not Hispanic

^b^Reference period: past 6 months

^c^Heroin, street fentanyl/carfentanil, prescription opioids, novel synthetics (i.e., U47700), buprenorphine, and/or methadone

^d^Use of ≥2 drug categories by any route in past 30 days (opioids, methamphetamine, cocaine/crack, prescription anxiety drugs, gabapentin, clonidine, and/or other)

^e^Injection drug use in past 30 days was an eligibility requirement for enrollment in WI

^f^Among participants who received a rapid HCV test (*n*=2,609 overall; IL: *n*=147; KY: *n*=337; NC: *n*=211; NE: *n*=547; OH: *n*=235; OR: *n*=159; WI: *n*=973)

^^^Subcategory of Opioids; percentages among all participants. Not all subcategories are shown

### Cohort description

Across the 7 ROI studies, the median baseline age was 34 years (interquartile range [IQR]: 28-42) (Table 2). The cohort included 57% men, 43% women, and 1% transgender participants and 83% were non-Hispanic white. Over half (53%) reported homelessness in the past 6 months, although this varied across studies (range: 36-68%). Overall, most participants identified opioids as their preferred drug for getting high (54%), however this varied across studies (range: 38-77%), followed by methamphetamines (36% overall: ranging from 4% in NE to over half of participants in OR and WI). Heroin was the most commonly preferred opioid (38%), followed by prescription opioids (9%), buprenorphine (3%), and fentanyl (2%). In the 30 days prior to interview, 86% of participants reported having used opioids, 36% reported having used fentanyl, 76% reported having used methamphetamine, 43% reported having used cocaine/crack, and 47% reported having used benzodiazepines. Polysubstance use was extremely common, with 85% of the overall sample reporting using multiple classes of drugs in the prior 30 days (median=3 drug classes). A large majority (92%) of participants reported ever injecting drugs, and across studies between 72% and >99% reported injection drug use in the past 30 days. Overall, missing data across key variables was low, ranging from 0% for age to 8.8% for HCV antibody status (see Supplemental Table 2 for missingness of key variables by study).

### Convergence and bottleneck plots

Convergence was not always achieved for the five key variables, as indicated by the last 25% of recruited participants having different characteristics than the first 75% (see Supplemental Figure 3 [study-specific convergence plots]). This pattern indicates that further sampling could have changed the prevalence estimates. For example, when comparing the estimated percentage in the first 75% of participants to the final complete group, homelessness in the past 6 months went from 40% to 55% in IL, past 30-day heroin use went from 57% to 47% in NC, HCV positive antibody status went from 62% to 57% in NE, methamphetamine as drug of choice went from 16% to 23% in OH and from 45% to 52% in WI, and heroin as drug of choice went from 39% to 34% in OR. In contrast, fentanyl for example converged quickly at multiple sites such as in WI, where past 30-day fentanyl use converged around 15% and remained consistent for most of study recruitment.

Bottlenecks were also present, with different recruitment chains converging on different prevalence estimates or failing to converge, even when the variable converged across the full sample (see Supplemental Figure 4 [study-specific bottleneck plots]). For example, past 30-day fentanyl use converged in the WI sample, however there were distinct bottlenecks by recruitment chain which failed to converge. Similarly, in KY, the longest recruitment chain converged to ~12.5%, but other chains did not converge or trended in different directions (i.e., one chain was approaching 50% and another approached 25%), suggesting that the recruitment chains may represent distinct subgroups, rather than the same underlying population.

### Degree and recruitment differences

Large degree ratios were present for some variables, indicating a potential for large weights to influence RDS-adjusted prevalence estimates (see Supplemental Table 3 [mean degree and degree differences]). Those with higher degrees tended to be younger, HCV antibody-positive, have experienced homelessness in the past 6 months, and have reported past 30-day heroin, fentanyl, and methamphetamine use. Recruitment trends did not always mirror trends in reported degree (see Supplemental Table 4 [mean number of recruits and recruitment ratios]).

### Recruitment homophily

Homophily for key variables varied across studies and was generally higher for age (range: 1.10 to 1.48 with age as a categorical variable) and drug of choice for getting high (range: 1.13 to 1.55) (Table 3). Homophily for past 30-day methamphetamine use was high in IL; homophily for past 30-day heroin use varied by study and was high for NC, OH, WI, OR, and IL, and past 30-day fentanyl use homophily was high only in NC and OH. The highest homophily for homelessness was reported in NE (1.18) and the highest homophily for HCV positive antibody status was reported in KY (1.18).

**Table 3:** Recruitment homophily, design effects and coefficient of discoverability for key variables by study

	**Study**						
	**IL**	**KY**	**NC**	**NE**	**OH**	**OR**	**WI**
**Homophily**							
Age (continuous/categorical)^a^	0.84/1.28	1.68/1.25	1.43/1.36	1.16/1.29	2.15/1.10	1.68/1.26	2.05/1.48
Heroin use^b^	1.44	1.10	1.27	1.02	1.21	1.28	1.23
Fentanyl use^b^	1.03	1.11	1.25	1.07	1.26	1.01	1.04
Methamphetamine use^b^	1.21	1.14	1.01	1.02	1.04	1.02	1.04
Preferred drug for getting high	1.55	1.35	1.51	1.13	1.25	1.38	1.42
HCV antibody status	1.16	1.18	1.01	1.11	1.08	1.01	1.11
Homelessness^c^	1.03	1.04	1.05	1.18	1.09	1.08	1.12
**Design Effects**							
Heroin use^b^	5.23	2.48	3.80	3.58	3.64	3.40	2.91
Fentanyl use^b^	4.97	2.06	3.59	2.79	2.68	1.52	2.08
Methamphetamine use^b^	2.06	1.92	3.04	2.80	2.93	1.97	2.05
HCV positive antibody status	5.42	2.46	3.22	2.58	2.99	3.21	2.67
Homelessness^c^	4.26	2.57	4.13	2.50	2.86	2.91	2.60
**Coefficient of discoverability**	1.12	0.90	1.29	1.28	1.05	1.02	1.27

*Abbreviations*: *IL* Illinois, *KY* Kentucky, *NC* North Carolina, NE New England (Massachusetts, New Hampshire, Vermont), *OH* Ohio, *OR* Oregon, *WI* Wisconsin

^a^Age categories: <25, 25-34, 35-44, 45-54, and ≥55

^b^Reference period: past 30 days

^c^Reference period: past 6 months

### Design effects

The average design effect varied by outcome of interest and by study. The average design effect across all outcomes and studies was 3.0. Design effects for past 30-day heroin use ranged from 2.48 (KY) to 5.23 (IL); past 30-day fentanyl use ranged from 1.52 (OR) to 4.97 (IL); past 30-day methamphetamine use ranged from 1.92 (KY) to 3.04 (NC); HCV positive antibody status ranged from 2.46 (KY) to 5.42 (IL); and homelessness (past 6 months) ranged from 2.50 (NE) to 4.26 (IL) (Table 3).

### Coefficients of discoverability

The coefficient of discoverability, or theta, estimates how much network size impacts the chance of being recruited. Theta was larger, at approximately 1.3, for NC, NE, and WI, suggesting that larger network size increased the speed of recruitment quite substantially for those with larger networks (Table 3). For IL, OH, and OR, theta was 1.12, 1.05, and 1.02 respectively, indicating that those with a larger network size were recruited moderately more quickly. Theta was <1.0 in KY, meaning that those with a larger network size were recruited into the study slower.

### Comparisons of RDS prevalence estimators and regression results

Prevalence estimates for all five key variables using RDS-I weights were similar to the unweighted prevalence estimates (Table 4). The largest differences in prevalence estimates were observed between unadjusted and RDS-II-adjusted estimates. This same pattern was seen in the fentanyl-HCV positive association estimates, with the unweighted and RDS-I weighted odds ratios/relative risks and corresponding confidence intervals being very close. The RDS-II weighted association was the most different and had the largest confidence intervals. RDS-II weights are based solely on degree and do not account for homophily, or cross-group recruitment, like the RDS-I estimator does; however, RDS-II degree weights are based on each individual’s degree and RDS-I degree weights are based on the average degree in a group. The use of tree-based bootstrapping and deletion of seeds from the dataset did not show any large or systematic change to the estimation of prevalence (Supplemental Table 5 [tree-based bootstrapping] Supplemental Table 6 [seed-bias analysis]). All three estimates of association were similar across all four weighting strategies in the same direction and similar magnitude (Table 5, Fig. 1 [forest plots of measures of association: relative risk], Supplemental Figure 5 [forest plots of measures of association: odds ratios], Supplemental Figure 6 [forest plots of measures of association by site: relative risk], Supplemental Table 7 [seed-bias analysis of association]).

**Table 4:** Comparison of unweighted and RDS-weighted prevalence estimates for key variables by study

	**Study**							
	**IL**				**KY**			
	**Unweighted Sample**	**RDS-I (Age)**	**RDS-I (Drug of Choice)**	**RDS-II**	**Unweighted Sample**	**RDS-I (Age)**	**RDS-I (Drug of Choice)**	**RDS-II**
**Heroin use**^a^	0.47 (0.40, 0.55)	0.46 (0.38, 0.54)	0.46 (0.38, 0.54)	0.43 (0.31, 0.56)	0.68 (0.63, 0.73)	0.67 (0.62, 0.72)	0.63 (0.57, 0.68)	0.57 (0.45, 0.68)
**Fentanyl use**^a^	0.26 (0.19, 0.33)	0.26 (0.19, 0.33)	0.26 (0.19, 0.32)	0.26 (0.15, 0.37)	0.31 (0.25, 0.36)	0.30 (0.25, 0.35)	0.26 (0.21, 0.31)	0.15 (0.07, 0.22)
**Methamphetamine use**^a^	0.80 (0.73, 0.86)	0.78 (0.71, 0.84)	0.79 (0.73, 0.86)	0.78 (0.69, 0.87)	0.79 (0.74, 0.83)	0.78 (0.74, 0.83)	0.75 (0.70, 0.80)	0.62 (0.50, 0.74)
**HCV antibody status**	0.45 (0.37, 0.53)	0.47 (0.38, 0.55)	0.44 (0.36, 0.52)	0.50 (0.37, 0.63)	0.62 (0.57, 0.67)	0.60 (0.55, 0.66)	0.59 (0.54, 0.65)	0.58 (0.47, 0.69)
**Homelessness**^b^	0.49 (0.41, 0.57)	0.47 (0.40, 0.55)	0.49 (0.41, 0.56)	0.58 (0.46, 0.69)	0.36 (0.31, 0.42)	0.37 (0.32, 0.42)	0.35 (0.30, 0.40)	0.36 (0.25, 0.47)
	**NC**				**NE**			
	**Unweighted Sample**	**RDS-I (Age)**	**RDS-I (Drug of Choice)**	**RDS-II**	**Unweighted Sample**	**RDS-I (Age)**	**RDS-I (Drug of Choice)**	**RDS-II**
**Heroin use**^a^	0.66 (0.61, 0.71)	0.64 (0.59, 0.70)	0.62 (0.57, 0.67)	0.48 (0.38, 0.57)	0.90 (0.88, 0.93)	0.90 (0.88, 0.93)	0.90 (0.88, 0.93)	0.89 (0.85, 0.92)
**Fentanyl use**^a^	0.49 (0.44, 0.55)	0.48 (0.42, 0.53)	0.45 (0.39, 0.51)	0.36 (0.26, 0.46)	0.68 (0.64, 0.71)	0.67 (0.63, 0.71)	0.67 (0.63, 0.71)	0.64 (0.60, 0.69)
**Methamphetamine use**^a^	0.93 (0.90, 0.96)	0.93 (0.90, 0.95)	0.92 (0.88, 0.95)	0.91 (0.85, 0.97)	0.36 (0.32, 0.40)	0.36 (0.32, 0.40)	0.36 (0.32, 0.40)	0.34 (0.29, 0.38)
**HCV antibody status**	0.65 (0.59, 0.72)	0.66 (0.59, 0.73)	0.63 (0.56, 0.70)	0.71 (0.62, 0.81)	0.59 (0.55, 0.64)	0.59 (0.55, 0.64)	0.59 (0.55, 0.63)	0.57 (0.52, 0.62)
**Homelessness**^b^	0.43 (0.38, 0.49)	0.43 (0.37, 0.48)	0.43 (0.37, 0.48)	0.35 (0.26, 0.44)	0.57 (0.53, 0.61)	0.56 (0.52, 0.60)	0.57 (0.53, 0.61)	0.52 (0.47, 0.56)
	**OH**				**OR**			
	**Unweighted Sample**	**RDS-I (Age)**	**RDS-I (Drug of Choice)**	**RDS-II**	**Unweighted Sample**	**RDS-I (Age)**	**RDS-I (Drug of Choice)**	**RDS-II**
**Heroin use**^a^	0.79 (0.73, 0.83)	0.78 (0.73, 0.83)	0.78 (0.73, 0.84)	0.77 (0.72, 0.83)	0.71 (0.60, 0.81)	0.60 (0.53, 0.68)	0.58 (0.51, 0.66)	0.55 (0.47, 0.63)
**Fentanyl use**^a^	0.63 (0.56, 0.69)	0.59 (0.52, 0.66)	0.62 (0.56, 0.68)	0.46 (0.35, 0.58)	0.12 (0.07, 0.17)	0.13 (0.07, 0.18)	0.10 (0.06, 0.15)	0.06 (0.02, 0.10)
**Methamphetamine use**^a^	0.80 (0.75, 0.85)	0.79 (0.73, 0.84)	0.79 (0.74, 0.84)	0.61 (0.48, 0.74)	0.97 (0.95, 1.00)	0.97 (0.94, 1.00)	0.97 (0.94, 1.00)	0.98 (0.96, 1.00)
**HCV antibody status**	0.71 (0.66, 0.77)	0.68 (0.61, 0.75)	0.71 (0.65, 0.77)	0.55 (0.42, 0.67)	0.53 (0.45, 0.61)	0.53 (0.45, 0.61)	0.51 (0.43, 0.59)	0.50 (0.34, 0.67)
**Homelessness**^b^	0.51 (0.45, 0.57)	0.50 (0.43, 0.56)	0.51 (0.44, 0.57)	0.43 (0.33, 0.54)	0.68 (0.61, 0.75)	0.67 (0.60, 0.75)	0.66 (0.58, 0.74)	0.55 (0.39, 0.71)
	**WI**							
	**Unweighted Sample**	**RDS-I (Age)**	**RDS-I (Drug of Choice)**	**RDS-II**				
**Heroin use**^a^	0.64 (0.61, 0.68)	0.63 (0.60, 0.67)	0.63 (0.60, 0.66)	0.60 (0.56, 0.65)				
**Fentanyl use**^a^	0.19 (0.17, 0.22)	0.19 (0.16, 0.21)	0.18 (0.16, 0.21)	0.16 (0.13, 0.19)				
**Methamphetamine use**^a^	0.91 (0.89, 0.93)	0.91 (0.89, 0.93)	0.91 (0.89, 0.93)	0.89 (0.87, 0.92)				
**HCV antibody status**	0.36 (0.33, 0.39)	0.36 (0.33, 0.39)	0.35 (0.32, 0.38)	0.34 (0.30, 0.38)				
**Homelessness**^b^	0.63 (0.60, 0.66)	0.63 (0.60, 0.66)	0.63 (0.60, 0.66)	0.59 (0.55, 0.64)				

Data presented as: estimate (95% CI)

*Abbreviations*: *IL* Illinois, *KY* Kentucky, *NC* North Carolina, *NE* New England (Massachusetts, New Hampshire, Vermont), *OH* Ohio, *OR* Oregon, *WI* Wisconsin

Prevalence estimates calculated using linear regression with robust confidence intervals

^a^Reference period: past 30 days

^b^Reference period: past 6 months

**Table 5:** Unweighted and RDS-weighted Relative Risk and Odds Ratio measures of association for the relationship between (a) fentanyl use, (b) heroin use, and (c) age and positive Hepatitis C Virus antibody status

	**(a) Fentanyl use**^**a**^** and** **HCV antibody status**		**(b) Heroin use**^**a**^** and** **HCV antibody status**		**(c) Age (per 10 years)**^b^** and** **HCV antibody status**	
***Relative Risk Regression***^1^	**RR**	**95% CI**	**RR**	**95% CI**	**RR**	**95% CI**
Unweighted	1.35	1.18, 1.54	1.44	1.28, 1.62	1.02	0.96, 1.09
**RDS-I:** Homophily by age	1.36	1.19, 1.55	1.44	1.30, 1.60	1.03	0.98, 1.08
**RDS-I:** Homophily by drug of choice	1.35	1.17, 1.56	1.44	1.28, 1.62	1.02	0.96, 1.09
**RDS-II:** Individual network size/degree	1.34	1.06, 1.70	1.31	1.06, 1.61	1.06	0.97, 1.15
***Logistic Regression***^2^	**OR**	**95% CI**	**OR**	**95% CI**	**OR**	**95% CI**
Unweighted	2.07	1.56, 2.74	2.13	1.77, 2.57	1.05	0.91, 1.20
**RDS-I:** Homophily by age	2.07	1.57, 2.72	2.12	1.75, 2.57	1.08	0.98, 1.20
**RDS-I:** Homophily by drug of choice	2.02	1.48, 2.76	2.10	1.74, 2.54	1.04	0.89, 1.21
**RDS-II:** Individual network size/degree	1.93	1.23, 3.02	1.87	1.37, 2.55	1.12	0.94, 1.33

*Abbreviations*: *CI* Confidence interval, *HCV* Hepatitis C virus, *OR* Odds ratio, *RR* Relative risk

^1^Relative risks estimated separately in each study using modified Poisson regression and combined across studies using random-effects meta-analyses

^2^Odds ratios estimated separately in each study using logistic regression and combined across studies using random-effects meta-analyses

^a^Reference period: past 30 days

^b^Age modeled as a continuous variable

## Discussion

RDS was used to successfully recruit PWUD from seven rural regions across the United States suggesting the potential for RDS to recruit individuals that are sometimes difficult to recruit. This paper advocates for comparing different RDS-estimators for this and other difficult to recruit populations, which may have large numbers of unproductive seeds and many short chains, as a way of evaluating the sensitivity of estimates to the different estimators. Some studies modified RDS recruitment procedures to meet enrollment goals, which could have impacted meeting RDS assumptions. For example, when seeds were unproductive or recruitment chains failed to produce additional recruits, studies enrolled additional seeds to reach the desired sample size. This additional seed recruitment resulted in many short recruitment chains (including 251 unproductive seeds and 376 seeds who only recruited one additional participant). Enrolling a large number of seeds can produce larger design effects (and higher homophily), prevent sample estimates from converging or reaching sample equilibrium, and increase the likelihood of observing bottlenecks which represent distinct sub-populations with different characteristics. Some studies permitted individuals to recruit up to 7 peers (although that was rare). Because individuals tend to recruit others who are more similar to themselves than to a randomly sampled individual from the target population, this could potentially increase homophily and result in larger design effects. Increasing the number of peer recruits per participant can also reduce the likelihood of achieving convergence on key variables because a majority of the sample is comprised of short chains, which individually have not reached sample equilibrium. Given the high homophily observed on some variables and lack of convergence on some key study variables, the RDS estimators used cannot adjust for biases introduced through sampling. That said, the proportionately large numbers of seeds and short chains may have reduced the impact of homophily. The initial sample may have been sufficiently diverse, i.e., seed bias was not detected, despite subsequent peer recruitments being correlated and demonstrating homophily on key variables. These patterns also may explain why tree bootstrapping did not outperform robust confidence intervals. As these patterns or outcomes were not predicted *a priori*, caution is warranted with respect to inference despite the overall recruitment success.

Recruitment differences across studies were observed on several key variables. Other unmeasured demographic, geographic, or outcome factors might also vary. Some of the observed differences were in opposite directions than the differences observed by degree, making it even more important to compare modeling strategies to see whether alternate modeling assumptions are impactful on the results. Of note, both RDS estimators used here include weights to account for differences in degree, but the RDS-I estimator additionally accounts for differences in cross-group recruitment. However, each RDS-I estimator accounts for differences in recruitment for only one variable at a time (i.e., differences in recruitment by age category or drug of choice), when recruitment differences and homophily were observed for multiple variables in the same study. As seen in the bottleneck plots, estimates across recruitment chains also varied and estimates within chains often did not converge, even when estimates across the larger sample did. In instances where bottlenecks are present and estimates are divergent for those in distinct recruitment chains, the standard recommendation would be to analyze these chains separately, as they likely represent distinct populations and not one completely connected to underlying population. For those variables where the estimates in the bottleneck appear to be converging to a common estimate, but estimates lack convergence in the same recruitment chain, the suggestion would be to continue recruitment until equilibrium is attained.

Furthermore, the presence of bottlenecks (i.e., different recruitment chains converge at different estimates) and the lack of cross-site recruitment within ROI studies, suggest that rather than sampling from one completely connected population, each RDS sample likely consists of multiple sub-populations. Simulation studies have demonstrated that these biases can be reduced when homophily is low and recruitment chains are long [29]; however, many RDS samples (similar to those observed here) consist of many short, wide recruitment chains, which may not be sufficient to remove seed bias and may also introduce bias related to differential recruitment behavior [29].

Bias can also be introduced when individuals preferentially recruit peers with similar characteristics (i.e., do not randomly recruit peers from their personal network) or the number of peer recruits differs by individual-level characteristics (i.e., differential recruitment success). This bias due to homophily can be particularly problematic when recruitment chains are short and wide rather than long and deep (i.e., few seeds, each recruiting a small number of peers, and recruitment chains which are sufficiently deep) and estimates for key characteristics have not converged (i.e., equilibrium has not been reached). A related bias, seed bias, occurs when the final sample is heavily influenced by the initial sample of seeds. For example, if most of the selected seeds use heroin and recruitment differs by heroin use (i.e., those who use heroin recruit more peers than those who do not use heroin), as well as high homophily on heroin (i.e., those who use heroin are more likely to recruit others who use heroin), the prevalence of heroin use in the resulting sample will overestimate the true population prevalence. Even when RDS weights are applied, it is possible for the RDS-I and RDS-II estimators to be biased by seed selection [14, 30], recruitment differences [15, 29, 31, 32], and bottlenecks [33].

Additionally, the large design effects observed suggest that the effective sample sizes are smaller and that estimates should account for the observed lack of independence resulting from peer recruitment. Other studies have similarly reported design effects ranging from 1.20 to 5.90 on key variables [23, 34, 35]. Of note, neither RDS estimator is designed to account for this lack of independence and failure to do so will result in artificially narrow confidence intervals [36]. The estimates presented here account for this lack of independence through the use of robust confidence intervals, although tree-based bootstraps were also considered.

Although several RDS estimators are available, none of the specific estimators are preferred in all instances. For example, the RDS-I estimator outperforms the RDS-II estimator when: [1] The seeds selected do not represent the underlying population; or [2] The sampling fraction is large and there is no differential recruitment. The RDS-II estimator can produce biased estimates in the presence of high homophily [29], differential recruitment [29], and large sampling fractions (>10%) [29, 31]. Because RDS-II weights are degree-based, estimates are sensitive to degree accuracy and differential degree [13, 37]. For example, if the mean degree is higher for individuals with attribute X and the sampling fraction is large, the prevalence of attribute X will likely be an underestimate due to the reliance on degree as a weight [32]. The RDS-II estimator can also lead to biased estimates in the presence of differential coupon rejection by peers and non-random recruitment of peers (based on characteristics of peer recruits); this bias is greatest when recruiters are more likely to avoid recruiting peers that they do not think would agree to participate (i.e., more likely to reject coupons) [13, 31]. Lu and colleagues also report a potential for biased estimates and larger standard errors, mean absolute errors, and design effects when participants preferentially recruit those they know better [31]. That said, we see compelling evidence that degree, as measured by the coefficient of discoverability, is a key factor in how quickly participants are recruited (Table 3).

Accurate estimates of the burden of opioid use, opioid and stimulant co-use, and polysubstance use in rural populations are needed to inform harm reduction and evidence-based treatment strategies to reduce opioid-related harms and increase evidence-based methods for substance use treatment. In these analyses, we present unweighted prevalence estimates, two different RDS-I-adjusted estimates, and RDS-II-adjusted estimates for five key variables in each of seven separately collected datasets. Although the RDS-adjusted estimates will not remove all potential sampling biases, the fact that estimates did not vary drastically across estimation approaches suggests that inferences (i.e., recommended interventions) would be similar regardless of the analytical approach used. Also, the above-described biases are less likely to impact measures of association than prevalence estimates and treatment of the data as a convenience sample is sensible.

Despite some of the limitations of the RDS sampling strategy noted above, these results shine a unique window into a difficult-to-reach population of high public health importance. Similar to our approach, future studies should consider presenting unweighted and RDS-adjusted estimates together, along with RDS diagnostics, so that a fuller understanding can be achieved. Future studies in rural areas may also benefit from conducting additional formative research prior to initiating RDS recruitment to better determine whether the underlying population is networked. If distinct subgroups exist, RDS may not be the most appropriate recruitment strategy.

## Conclusion

Conducting research in hard-to-reach, marginalized populations requires carefully applied recruitment techniques that can be challenging to implement. RDS was used to successfully recruit PWUD from seven rural U.S. regions. Despite its limitations, RDS has recruitment advantages over other approaches, which are primarily location-based or rely on outreach workers. We have described how RDS-adjusted prevalence estimators perform in a series of rural studies on the use of non-prescribed opioids and how failure to meet key RDS assumptions impacts their performance. Understanding which, if any, RDS assumptions are not met is critical and provides important insights necessary to interpret how the resulting estimates may be biased. However, at the same time, the range of variation across these different studies is reassuringly limited. This includes variability in the direction and strength of each association under different weighting schemes. That said, there may still be an advantage to presenting a range of estimators for prevalence estimates, to show sensitivity to different weighting assumptions and to use care in the interpretation of such estimates.

### Supplementary Information

**Supplementary Material 1.** 

**Supplementary Material 2.** 

**Supplementary Material 3.** 

**Supplementary Material 4.** 

**Supplementary Material 5.** 

**Supplementary Material 6.** 

**Supplementary Material 7.** 

## Data Availability

The datasets generated and/or analyzed during the current study are not publicly available due the sensitive nature of the topic area. However, ROI data are available on reasonable request. Please see the ROI website (https://ruralopioidinitiative.org) for more information on how to complete a concept proposal and data request. Please contact the ROI consortium (ruralopioids@uw.edu) or Heidi Crane (hcrane@uw.edu) for more information or for data requests.

## Abbreviations

ACASI: Audio Computer-Assisted Self-Interviewing
ARC: Appalachian Regional Commission
CDC: Centers for Disease Control and Prevention
CI: Confidence intervals
HCV: Hepatitis C virus
HRS: Harm reduction services
IDU: Injection drug use
IL: Illinois
KY: Kentucky
NC: North Carolina
NE: New England
OH: Ohio
OR: Oregon
OR: Odds ratio
NIDA: National Institute on Drug Abuse
PWUD: Persons who use drugs
RDS: Respondent-driven sampling
ROI: Rural Opioid Initiative
RR: Relative risk
SAMHSA: Substance Abuse and Mental Health Services Administration
WI: Wisconsin
WV: West Virginia
