# Supplementary material for: Evaluation of respondent-driven sampling in seven studies of people who use drugs from rural populations: findings from the Rural Opioid Initiative
Source: BMC Med Res Methodol. 2024 Apr 23;24:94. doi: 10.1186/s12874-024-02206-5 (PMC11036624; doi:10.1186/s12874-024-02206-5)
Supplement: Supplementary file 1 — Supplementary Material 1. [file 12874_2024_2206_MOESM1_ESM.docx]

**Supplemental Figure 1.** Location of studies in the Rural Opioid Initiative.


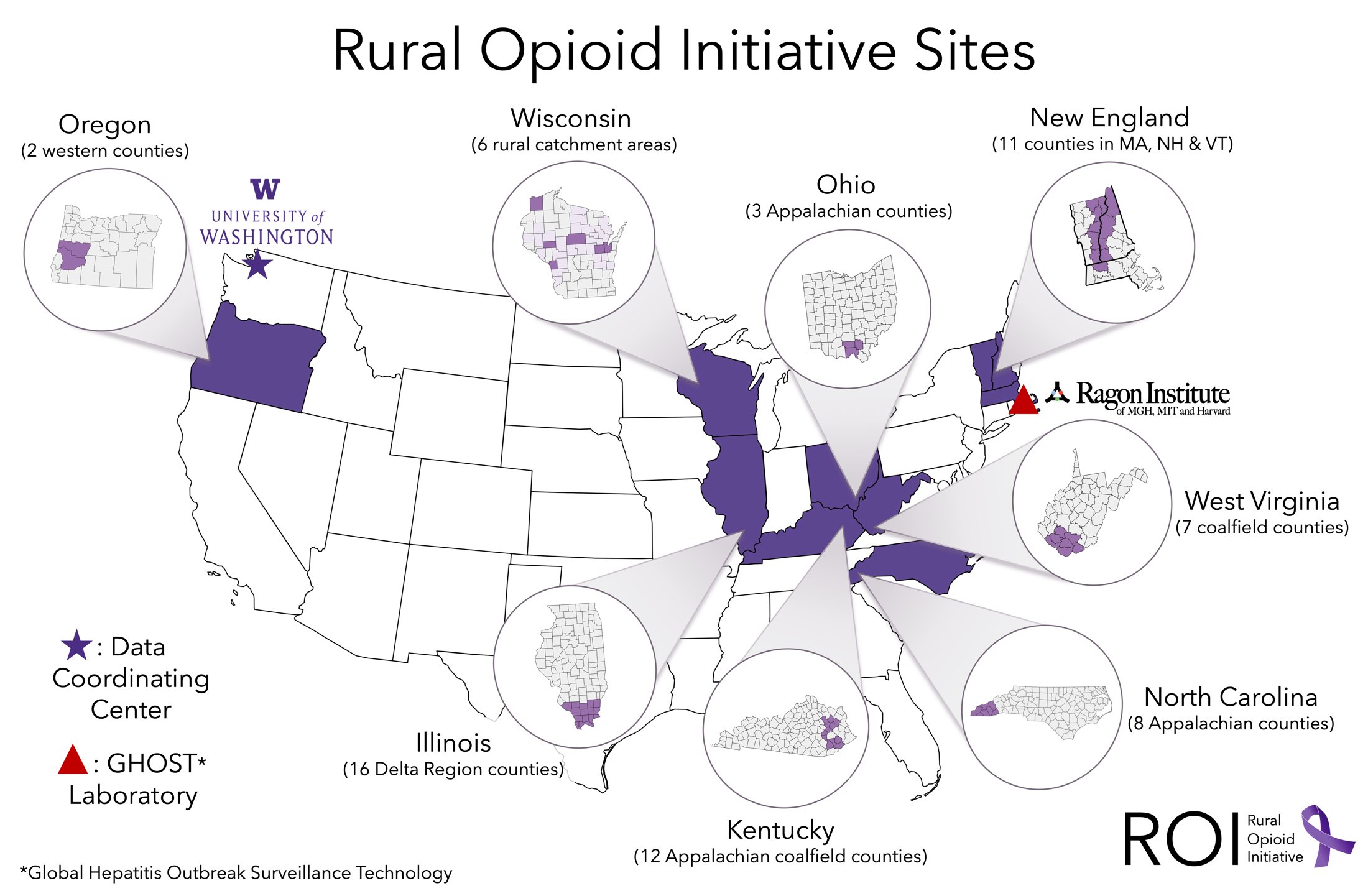


Abbreviations: MA, Massachusetts; NH, New Hampshire; VT, Vermont.

Note Supplemental Figure 1 was previously published in Jenkins et al., *Addict Sci Clin Pract.* 2022;17(1):38. https://pubmed.ncbi.nlm.nih.gov/35883197/
