# Supplementary material for: Evaluation of respondent-driven sampling in seven studies of people who use drugs from rural populations: findings from the Rural Opioid Initiative
Source: BMC Med Res Methodol. 2024 Apr 23;24:94. doi: 10.1186/s12874-024-02206-5 (PMC11036624; doi:10.1186/s12874-024-02206-5)
Supplement: Supplementary file 4 — Supplementary Material 4. [file 12874_2024_2206_MOESM4_ESM.docx]

**Supplemental Figure 4.** Bottleneck plots by study.

1. **Illinois**

1. **Kentucky**


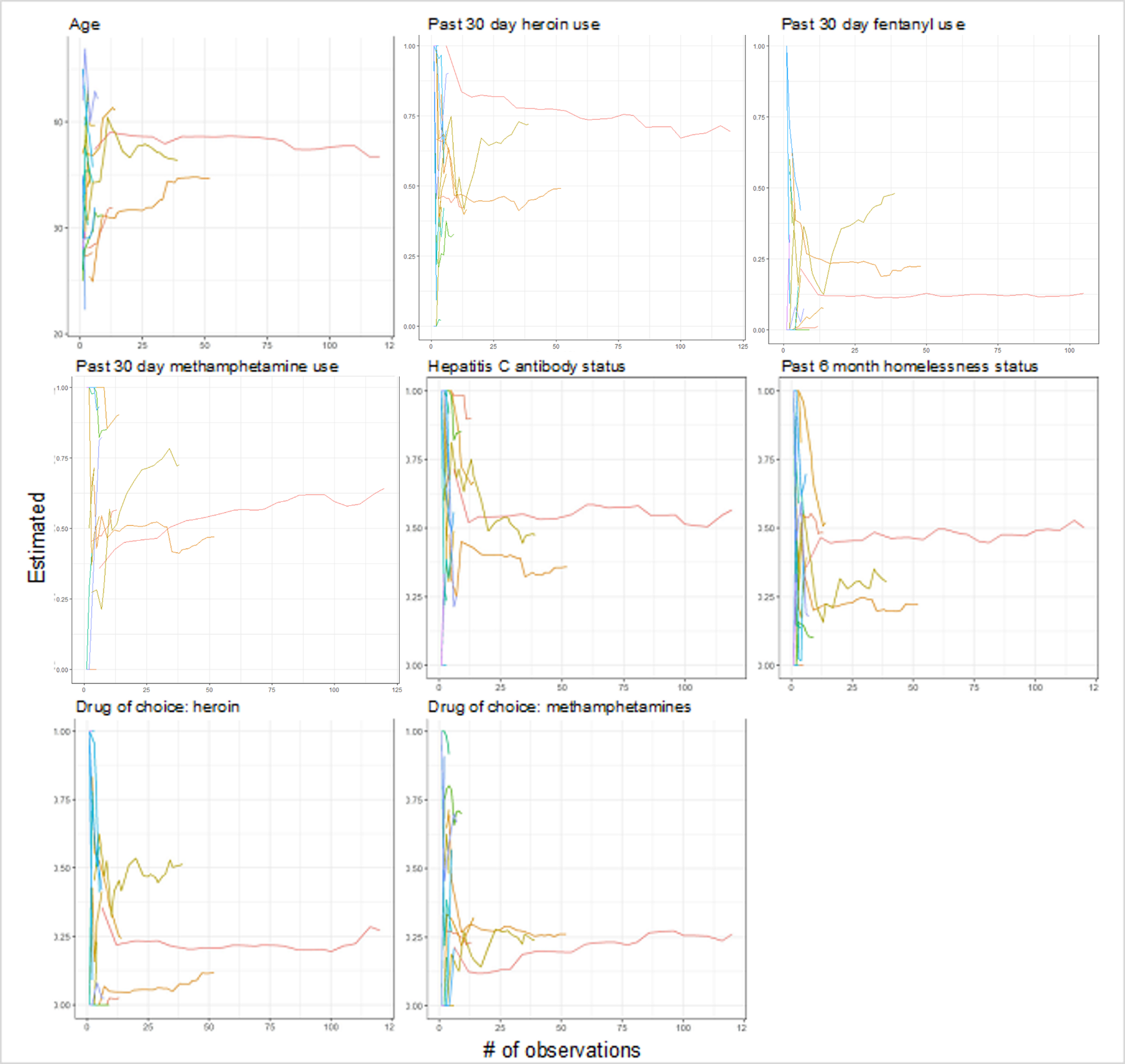


1. **North Carolina**

1. **New England**

1. **Ohio**

1. **Oregon**

1. **Wisconsin**
