# Supplementary material for: Evaluation of respondent-driven sampling in seven studies of people who use drugs from rural populations: findings from the Rural Opioid Initiative
Source: BMC Med Res Methodol. 2024 Apr 23;24:94. doi: 10.1186/s12874-024-02206-5 (PMC11036624; doi:10.1186/s12874-024-02206-5)
Supplement: Supplementary file 6 — Supplementary Material 6. [file 12874_2024_2206_MOESM6_ESM.docx]

**Supplemental Figure 6.** Forest plots of unweighted and RDS-weighted Relative Risk measures of association for the relationship between (a) fentanyl use, (b) heroin use, and (c) age and positive Hepatitis C Virus antibody status by site

1. **Fentanyl use**^a^ **and HCV antibody status**


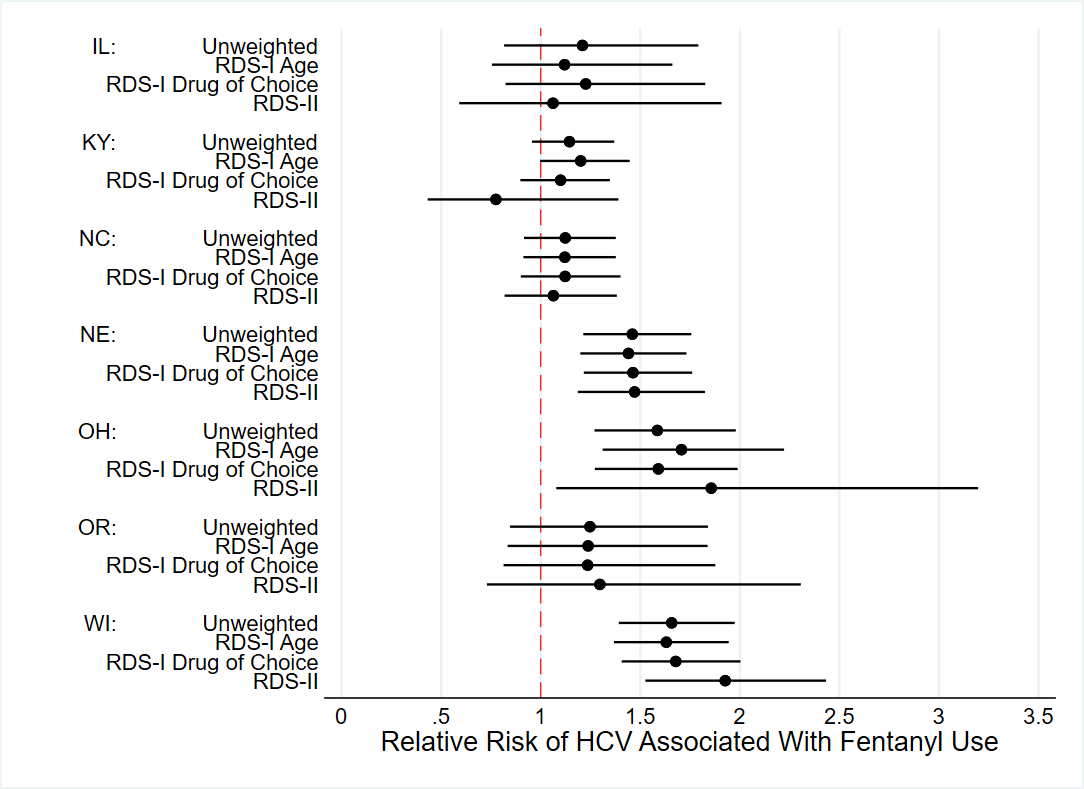


1. **Heroin use**^a^ **and HCV antibody status**


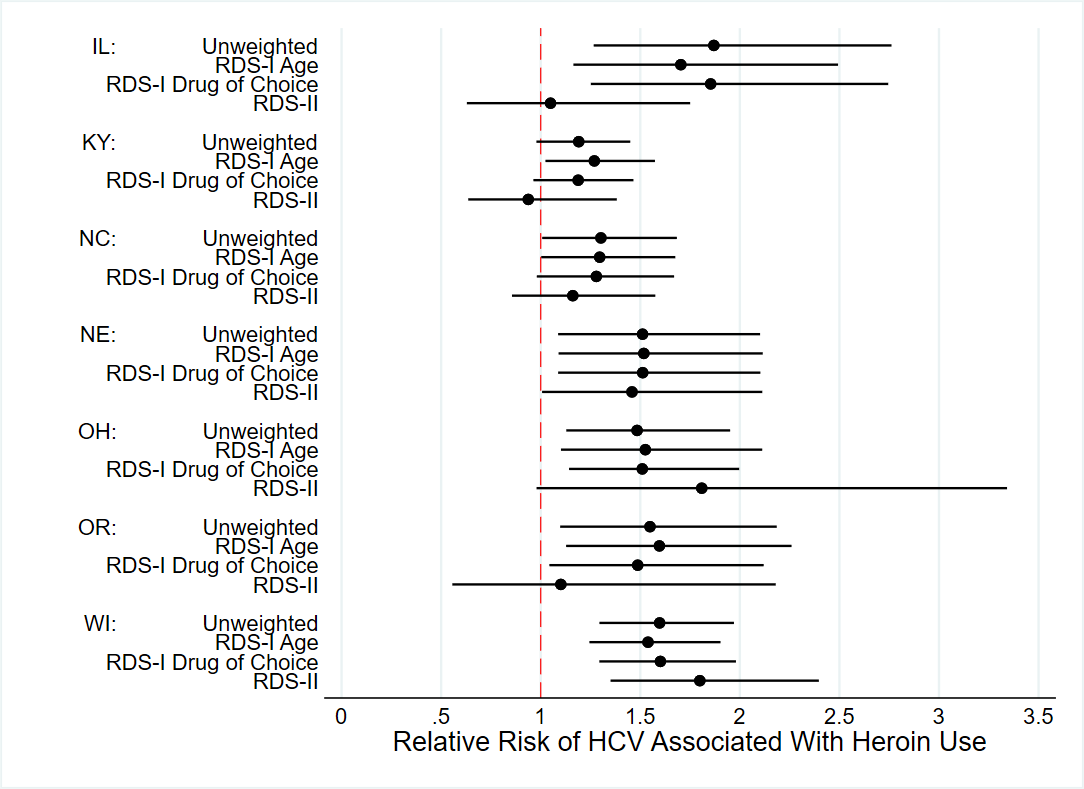


1. **Age and HCV antibody status**


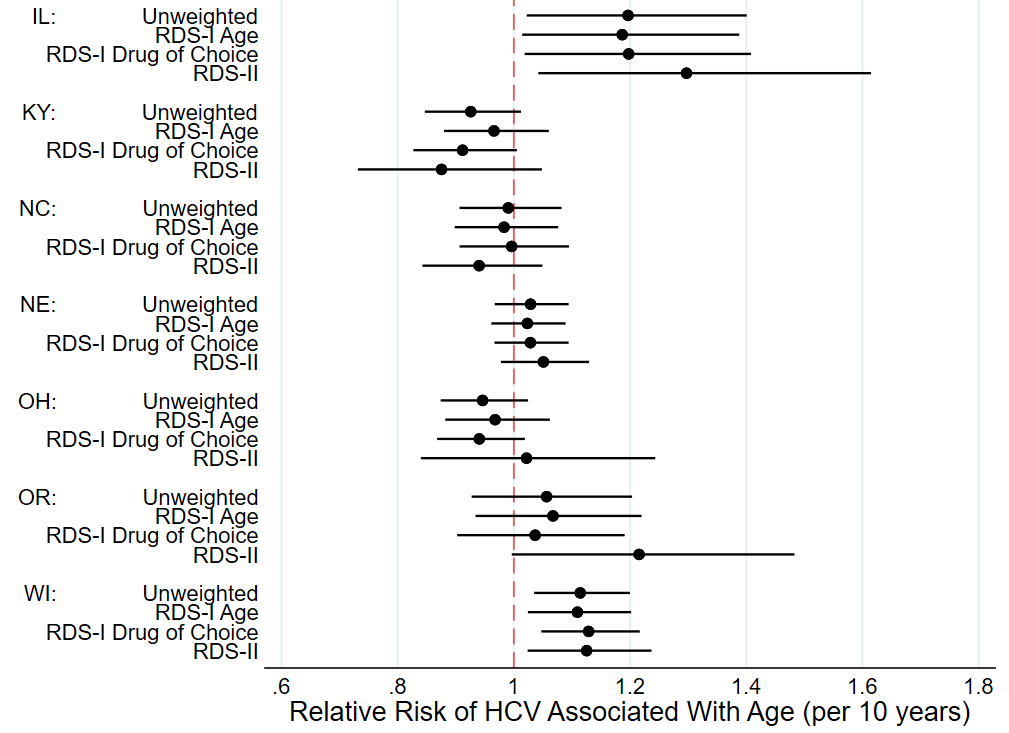


Study abbreviations: IL, Illinois; KY, Kentucky; NC, North Carolina; NE, New England (Massachusetts, New Hampshire, Vermont); OH, Ohio; OR, Oregon; WI, Wisconsin.

Abbreviations: HCV, hepatitis C virus.

^a^ Reference period: past 30 days.
