# Supplementary material for: Evaluation of respondent-driven sampling in seven studies of people who use drugs from rural populations: findings from the Rural Opioid Initiative
Source: BMC Med Res Methodol. 2024 Apr 23;24:94. doi: 10.1186/s12874-024-02206-5 (PMC11036624; doi:10.1186/s12874-024-02206-5)
Supplement: Supplementary file 7 — Supplementary Material 7. [file 12874_2024_2206_MOESM7_ESM.docx]

**Supplemental Table 1.** STROBE RDS checklist.

| **Item** | **#** | **Complete STROBE-RDS Checklist** | **Included** | **Page(s)** | **Note(s)** |
| --- | --- | --- | --- | --- | --- |
| **Title and Abstract** | 1 | (a) Indicate “respondent-driven sampling" in the title or abstract | ☑ | 1 |  |
|  |  | (b) Provide in the abstract an informative and balanced summary of what was done and what was found | ☑ | 7 |  |
| **Introduction** |  |  |  |  |  |
| *Background/ rationale* | 2 | Explain the scientific background and rationale for the investigation being reported | ☑ | 9-11 |  |
| *Objectives* | 3 | State specific objectives | ☑ | 11 |  |
| **Methods** |  |  |  |  |  |
| *Study design* | 4 | (a) Present key elements of study design early in the paper | ☑ | 12-13 |  |
|  |  | (b) State why RDS was chosen as the sampling method | ☑ | 9-11 |  |
| *Setting* | 5 | a) Describe the setting, locations, and relevant dates, including periods of recruitment, and data collection | ☑ | 12 | [See study-specific details below.](#Setting5a) |
|  |  | (b) Describe formative research findings used to inform RDS study | ⮽ | — | — |
| *Participants* | 6 | (a) Give the eligibility criteria, and the sources and methods of selection of participants. Describe how participants were trained/ instructed to recruit others, number of coupons issued per person, any time limits for referral | ☑ | 12-13 | [See study-specific details below.](#Participants6a) |
|  |  | (b) Describe methods of seed selection and state number at start of study and number added later. State when and how additional seeds were recruited | ☑ | 12-13 and  Table 1 | [See study-specific details below.](#Participants6b) |
|  |  | (c) State if there was any variation in study procedures during data collection (e.g., changing numbers of coupons per recruiter, interruptions in sampling, or stopping recruitment chains) | ☑ | — | [See study-specific details below.](#Participants6c) |
|  |  | (d) Report wording of personal network size question(s) | ☑ | — | [See study-specific details below.](#Participants6d) |
|  |  | (e) Describe incentives for participation and recruitment | ☑ | 13 and  Table 1 | [See study-specific details below.](#Participants6e) |
| *Variables* | 7 | (a) If applicable, clearly define all outcomes, predictors, and diagnostic criteria | ☑ | 13-15 |  |
|  |  | (b) State how recruiter-recruit relationship was tracked | ☑ | — | [See study-specific details below.](#Variables7b) Additionally, each participant who returned a coupon was asked the following question:  *If someone recruited you for this study, how do you know them?*   1. *Partner, spouse, boyfriend, girlfriend* 2. *Casual sex partner* 3. *Friend, associate, acquaintance* 4. *Family member* 5. *Neighbor* 6. *Person I use drugs with* 7. *Service or program staff* 8. *Stranger* 9. *Other* |
| *Data sources/ measurement* | 8 | (a) For each variable of interest, give sources of data and details of methods of measurement. | ☑ | 13 |  |
|  |  | (b) Describe methods to assess eligibility and reduce repeat enrolment (e.g., coupon manager software, biometrics) | ☑ | — | [See study-specific details below.](#Measures8b) |
| *Bias* | 9 | Describe any efforts to address potential sources of bias | ☑ | — | [See study-specific details below.](#Bias9) |
| *Study size* | 10 | Explain how the study size was arrived at | ☑ | — | [See study-specific details below.](#StudySize10) |
| *Quantitative variables* | 11 | Explain how quantitative variables were handled in the analyses. If applicable, describe which groupings were chosen, and why | ☑ | 13-15 |  |
| *Statistical methods* | 12 | (a) Describe all statistical methods, including those to account for sampling strategy (e.g., the estimator used) | ☑ | 13-15 |  |
|  |  | (b) State data analysis software, version number and specific analysis settings used | ☑ | 15 |  |
|  |  | (c) Explain how missing data were addressed | ☑ | 15 |  |
|  |  | (d) Describe any sensitivity analyses | ☑ | 13-15 | Sensitivity analyses included: (1) a comparison of the tree bootstrap approach of Baraff *et al.* vs. use of robust confidence intervals; (2) seed-bias analyses removing seeds; and (3) a comparison of the magnitude and direction of effect estimates of the associations when RDS sampling weights were applied or not using relative risk regression and logistic regression with pooled estimates. |
|  |  | (e) Report any criteria used to support statements on whether estimator conditions or assumptions were appropriate | ☑ | 13-15 |  |
|  |  | (f) Explain how seeds were handled in analysis | ☑ | 13-14 |  |
| **Results** |  |  |  |  |  |
| *Participants* | 13 | a) Report the numbers of individuals at each stage of the study — e.g., numbers potentially eligible, examined for eligibility, confirmed eligible, included in the study, and analyzed | ⮽ | — | — |
|  |  | (b) Give reasons for non-participation at each stage (e.g., not eligible, does not consent, decline to recruit others) | ⮽ | — | — |
|  |  | (c) Consider use of a flow diagram | ⮽ | — | — |
|  |  | (d) Report number of coupons issued and returned | ☑ | Table 1 | — |
|  |  | (e) Report number of recruits by seed and number of RDS recruitment waves for each seed. Consider showing graph of entire recruitment network | ☑ | Supplemental Figure 2 |  |
|  |  | (f) Report recruitment challenges (e.g., commercial exchange of coupons, imposters, duplicate recruits) and how addressed | ☑ | — | [See study-specific details below.](#Participants13f) |
|  |  | (g) Consider reporting estimated design effect for outcomes of interest | ☑ | Table 3 |  |
| *Descriptive data* | 14 | a) Give characteristics of study participants (e.g., demographic, clinical, social) Report unweighted sample size and percentages, estimated population proportions or means with estimated precision (e.g., 95% confidence interval) | ☑ | Table 2,  Table 4 |  |
|  |  | (b) Indicate the number of participants with missing data for each variable of interest | ☑ | Supplemental Table 2 |  |
| *Outcome data* | 15 | If applicable, report number of outcome events or summary measures | ☑ | Table 2 |  |
| *Main results* | 16 | (a) Give unadjusted and study design adjusted estimates and their precision (e.g., 95% confidence intervals). | ☑ | Table 4,  Table 5,  Supplemental Tables 5-7, Supplemental Figure 5 |  |
|  |  | (b) Report category boundaries when continuous variables were categorised | ☑ | 16 | Age was the only continuous variable that was categorised. |
|  |  | (c) If adjustment of primary outcome leads to marked changes, report information on factors influencing the adjustments (e.g., personal network sizes, recruitment patterns by group) | ☑ | 19-20 |  |
| *Other analyses* | 17 | Report other analyses done—e.g., sensitivity analyses, different RDS estimators and definitions of personal network size | ☑ | 19-20  Table 4,  Table 5,  Supplemental Figure 4, Supplemental Tables 5-7 | Sensitivity analyses included: (1) a comparison of the tree bootstrap approach of Baraff *et al.* vs. use of robust confidence intervals; (2) seed-bias analyses removing seeds; and (3) a comparison of the magnitude and direction of effect estimates of the associations when RDS sampling weights were applied or not using relative risk regression and logistic regression with pooled estimates. |
| **Discussion** |  |  |  |  |  |
| *Key results* | 18 | Summarise key results with reference to study objectives | ☑ | 20-21 |  |
| *Limitations* | 19 | Discuss limitations of the study, taking into account sources of potential bias or imprecision. Discuss both direction and magnitude of any potential bias | ☑ | 20-23 |  |
| *Interpretation* | 20 | Give a cautious overall interpretation of results considering objectives, limitations, multiplicity of analyses, results from similar studies, and other relevant evidence | ☑ | 20-23 |  |
| *Generalisability* | 21 | Discuss the generalisability (external validity) of the study results | ☑ | 20-23 |  |
| **Other information** |  |  |  |  |  |
| *Funding* | 22 | Give the source of funding and the role of the funders for the present study and, if applicable, for the original study on which the present article is based | ☑ | 27-29 |  |

| **Item** | **#** | **IL** | **KY** | **NC** | **NE** | **OH** | **OR** | **WI** |
| --- | --- | --- | --- | --- | --- | --- | --- | --- |
| **Methods** |  |  |  |  |  |  |  |  |
| *Setting* | 5a | Setting/location: 16 Delta Region counties in southern Illinois. | Setting/location: 5 Appalachian counties in eastern Kentucky (12 Appalachian Counties total with survey data collection from people who used drugs focused on 5 of the 12 counties). | Setting/location: 8 Appalachian counties in western North Carolina. | Setting/location: 3 regions (Tristate, Central, and Northern) composed of 11 rural counties in central Massachusetts (1), eastern Vermont (6), and western New Hampshire (4). | Setting/location: 3 Appalachian counties in southern Ohio. | Setting/location: 2 rural counties in western Oregon. | Setting/location: 6 rural catchment areas (service areas of local harm reduction service (HRS) organization) in rural central and northern Wisconsin. |
|  |  | Relevant dates: Recruitment and data collection occurred between July 2018 and July 2019. | Relevant dates: Recruitment and data collection occurred between February 2018 and March 2020. | Relevant dates: Recruitment and data collection occurred between February 2019 and March 2020. | Relevant dates: Recruitment and data collection occurred between May 2018 and October 2019. | Relevant dates: Recruitment and data collection occurred between March 2019 and December 2019. | Relevant dates: Recruitment and data collection occurred between March 2018 and April 2019. | Relevant dates: Recruitment and data collection occurred between January 2018 and July 2019. |
|  |  | Venues: 1 mobile HRS organization unit which recruited participants during service delivery, as well as 2 static sites run by same HRS. Participants were also recruited at pop up cookouts in public settings (e.g., gas stations, health fairs, etc.) as well as in retail locations (e.g., pawn shops, etc.) and homeless shelters (16 pop-up events total). | Venues: 1 static site (centrally located store-front, field office). Participants were recruited through: (1) a previous online study of young people who use drugs (PWUD) in the study area who consented to be contacted about future research; or (2) targeted outreach that involved distributing flyers at local businesses and organizations, as well as hosting community cookouts that advertised the study. | Venues: 2 static sites located within county health departments (Jackson and Cherokee counties). Flyers were posted in several local gas station/ convenience stores, in the health department waiting areas, and several other fixed locations. | Venues: 11 static sites, 9 of which were co-located near or within HRS organizations, medical centers, community health centers, churches, and/or homeless shelters. A majority (9 of 11) of the sites were in the town center or a short walk from the local town center; the other 2 sites were in busy areas within 15-20 minutes of town center. | Venues: 4 static sites (1 within a local health department (LHD) SSP, 1 within a faith-based outreach program, and 2 within social services or treatment-focused locations), as well as 1 mobile tabling site at different convenience store locations. Study flyers were also distributed by study staff in numerous public settings (e.g., gas stations, treatment centers, healthcare settings, churches, and food banks [~30-40 locations in total]), as well as shared through stakeholder organization websites and through social media outlets in the community, as appropriate. | Venues: 2 static community-based organization (CBO) sites (HIV Alliance and Community Center) which recruited participants during service delivery. Mobile recruitment was conducted in public outdoor locations throughout both counties. | Venues: 6 static HRS organization sites (formerly AIDS Resource Center of Wisconsin, now Vivent Health) which recruited participants during service delivery. |
| *Participants* | 6a | Overall eligibility criteria (for all participants): Age 15 or older; current resident of the study area; English speaking; past 30-day use of opioids (any route of administration) OR injection drug use of any type “to get high” in the past 30 days. | Overall eligibility criteria (for all participants): Age 18 or older; current resident of the study area; past 30-day use of opioids (any route of administration) OR injection drug use of any type  “to get high” in the past 30 days. | Overall eligibility criteria (for all participants): Age 18 or older; current resident of the study area (based on zip code); past 30-day use of opioids (any route of administration) OR injection drug use of any type “to get high” in the past 30 days. | Overall eligibility criteria (for all participants): Age 18 or older; current resident of the study area (based on zip code); English speaking; past 30-day use of opioids (any route of administration) OR injection drug use of any type “to get high” in the past 30 days. | Overall eligibility criteria (for all participants): Age 18 or older; current resident of the study area; past 30-day use of opioids (any route of administration) OR injection drug use of any type “to get high” in the past 30 days. | Overall eligibility criteria (for all participants): Age 18 or older; current resident of the study area; English speaking; past 30-day recreational opioid use (any route of administration) OR injection drug use of any type “to get high” in the past 30 days. | Overall eligibility criteria (for all participants): Age 15 or older; current resident within the service areas of the HRS organization sites in rural central and northern WI; injection drug use of any type “to get high” in the past 30 days. |
|  |  | Coupons: Each person received 6 coupons to distribute to peers. Coupons had an expiration date of 3 months (this was tapered to 1 month near the end of the recruitment period). | Coupons: Each person received 3 coupons to distribute to peers. Coupons had an expiration date of 2 weeks, but this was not enforced. | Coupons: Each person received 3 coupons to distribute to peers. However, when recruitment was slow, some participants (n=18) received an additional coupon (for a total of 4). Coupons had an expiration date of 4 weeks, but this was not enforced. (Note: see exceptions in [section 6c](#NC6c)) | Coupons: Each person received 3 coupons to distribute to peers. Coupons had an expiration date of 2 weeks, but this was not enforced. (Note: see exceptions in [section 6c](#NE6c)) | Coupons: Each person received up to 4 coupons to distribute to peers.  (Note: see exceptions in [section 6c](#OH6c)) | Coupons: Each person received 3 coupons to distribute to peers. Coupons did not expire. (Note: see exceptions in [section 6c](#OR6c)) | Coupons: Each person received 3 coupons to distribute to peers. Upon successful referral of 3 people for screening, participants we provided 2 additional coupons (for a total of 5). |
|  |  | Recruitment training: Study staff covered tips on how to successfully recruit peers. | Recruitment training: Study staff covered tips on how to successfully recruit peers. | Recruitment training: Study staff asked participants to think about who they were going to give the coupons to and what they would say when they handed out a coupon. | Recruitment training: Study staff reviewed the coupon-based RDS recruitment and referral component with seeds. | Recruitment training: Study staff provided participants with training in the handling of the peer recruitment coupons and the recruitment process. Participants were instructed not to pressure their peers into participating in the study. Interested peers received the referral coupon and a description of the study from the initial participant and called for an appointment or presented themselves at the survey office. | Recruitment training: Study staff instructed seeds to recruit people they know and have seen in the past 30 days and who inject drugs or use opioids. | Recruitment training: Study staff covered tips on how to successfully recruit peers. |
|  | 6b | Additional seed eligibility (apart from overall eligibility): None. | Additional seed eligibility (apart from overall eligibility): Same for seeds and peer recruits, with the exception that peer recruits did not have to meet the criteria for having a big network size. To qualify as seeds, participants had to meet additional eligibility criteria and be “highly connected,” defined as reporting having used drugs with ≥10 people in the past 30 days for women and ≥20 people in the past 30 days for men.^a^ | Additional seed eligibility (apart from overall eligibility): None. | Additional seed eligibility (apart from overall eligibility): None. | Additional seed eligibility (apart from overall eligibility): None. | Additional seed eligibility (apart from overall eligibility): None. | Additional seed eligibility (apart from overall eligibility): No additional requirements, however field staff were trained to find individuals that were dynamic, knowledgeable about and well connected to PWUD, and motivated to recruit new participants. |
|  |  | Seed recruitment/ enrollment: Seeds were enrolled on a rolling basis. Study staff, with assistance from the HRS provider, attempted to identify individuals who were well linked socially and/or who were outspoken advocates of their community. Additionally, anyone who was not directly referred into the study via a coupon was considered a seed. | Seed recruitment/ enrollment: Seeds were enrolled on a rolling basis. | Seed recruitment/ enrollment: Seeds were enrolled on a rolling basis. Both field sites started with 3 seeds, and seeds were added whenever recruitment slowed. | Seed recruitment/ enrollment: Seeds were enrolled on a rolling basis. Replacement seeds were recruited if no referrals came in from an initial seed by 3 days after the 2-week follow-up from the study visit. Seeds at each of the study sites were identified through referrals from HRS agencies, as well as recruitment from flyers, social media, web-based advertising, and street outreach; most seeds were recruited through street outreach. | Seed recruitment/ enrollment: Seeds were enrolled on a rolling basis. Seeds at each of the study sites were identified with the help of local stakeholders, including SSP staff and other individuals connected to PWUD, as well as recruitment from flyers, social media, web-based advertising, and street outreach. Potential seeds were also identified through qualitative interviews with PWUD conducted by study staff in the area; PWUD who participated in an in-depth interview, were interested in being contacted again for participation in the survey, and gave consent for re-contact, were re-contacted for the study. | Seed recruitment/ enrollment: Seeds were enrolled on a rolling basis. The study began with 10 seeds and added seeds incrementally to speed recruitment during dips in referrals. Seed recruitment occurred in the context of SSPs and field outreach. Peer recovery specialists based in the study communities helped to select seeds who appeared to be well-connected to other PWUD (including secondary exchangers of syringe supplies). Study staff tracked demographics and drug of choice closely during recruitment and prioritized seed recruitment of underrepresented groups when indicated. | Seed recruitment/ enrollment: Seeds were enrolled on a rolling basis. Each site began with 1 seed and recruited an additional 2 each week if the initial seed was not productive. Field staff were trained to find individuals that were dynamic, knowledgeable about and well connected to PWUD, and motivated to recruit new participants. Considerations for seed recruitment: (1) demographic/drug use diversity (e.g., age, gender, drugs used, etc.); (2) geographic diversity; (3) network characteristics; (4) focus on those using opioids who may have injected in the past. |
|  |  | # of seeds: 53 | # of seeds: 48 | # of seeds: 50 | # of seeds: 51 | # of seeds: 45 | # of seeds: 42 | # of seeds: 273 |
|  | 6c | There were no changes to coupon numbers or stopped recruitment chains. | After the first year of recruitment, 1 county accounted for 65% of the sample (n=266 at the end of Year 1) although recruitment efforts were equitable across all counties. To increase enrollment from the 4 under-represented counties, staff scaled up targeted outreach efforts in those counties and stopped outreach in the primary county (stopped enrolling people with some rare exceptions) in January 2019. After the first year of recruitment, only 15% of the sample came from the county most represented in Year 1. | In December 2019, when recruitment slowed, we received IRB approval to recontact productive recruiters and offer them 1 additional coupon, increasing the number of coupons each participant received from 3 to 4; this resulted in 19 people receiving 4 coupons instead of 3. | Initially, inclusion criteria required that participants were “not currently enrolled in addiction treatment”; this criterion was dropped after finding that there were several potential participants who were enrolled in a treatment program but who disclosed drug use in the past 30 days and indicated poor adherence to treatment and continued use of opioids.  In July 2018, the number of coupons provided to recruiters during data collection was changed. Any participant for whom all 3 initial coupons were returned was given up to 4 additional recruitment coupons (maximum 7); 31 participants were enrolled before the change and 558 participants were enrolled after the change. (Note: 3 participants mistakenly received 5 additional coupons and 4 participants mistakenly received 6 additional coupons; this was a result of participants reporting lost coupons that were replaced by study staff and then ended up being returned. No participant received payment for more than 7 referrals.)  Recruitment at 1 site was temporarily halted for ~6 weeks due to the need to find alternative office space when the initial study site offices proved to be inadequate to accommodate the project. Recruitment chains were continued after the interruption. | Participants were initially given 5 coupons each, however in June 2019 this had to be revised down to 4 coupons in order to stay below an IRB-imposed limit on the amount of gift card reimbursement allowed per study participant per year for a single study; n=163 participants were enrolled prior to the change and n=98 participants were enrolled after. | In July 2018, after a seed recruited a third participant for the first time, a change was made to allow seeds with 3 successful recruits to recruit a 4th person. At the point of this change, all participants – regardless of when enrolled – were informed that they were permitted to recruit 4 participants, since the original IRB submission allowed for this possibility. | In 1 county, a student enrolled more than 3 seeds a week during the months of June to August 2018. |
|  | 6d | Please think about the people living in the southern 16 Illinois counties who are at least 15 years of age and who to your knowledge, have used heroin or prescription opioids (through any route of administration), or who have injected any type of drug to get high in the past 30 days. How many of these people do you know personally? By this, I mean people that you know and who also know you. | Please think about the people living in A, B, C, D, and/or E County who are at least 18 years of age and who to your knowledge, have used heroin or prescription opioids (through any route of administration) or have injected any type of drug to get high in the past 30 days. How many of these people do you know personally? These people should be people who you know and who also know you. | Please think about people living in the area who to your knowledge, have injected any type of drug or have used opioids to get high in the past 30 days. How many of these people do you know personally? By this, I mean people that you know and who also know you. | The personal network size question (below) was not added until December 2018, resulting in only ~40% of participants with responses. Therefore, network size was derived for each participant using social network and coupon data.  Please think about the people living in the area who are at least 18 years of age and who to your knowledge have injected drugs or used heroin or opiate painkillers to get high in the past 30 days. How many of these people do you know personally? By this, I mean people that you know, who also know you. | Please think about the people living in your county who are at least 18 years of age and who, to your knowledge, have used heroin or prescription opioids (through any route of administration), or who have injected any type of drug in the past 30 days. How many people do you know personally? By this, I mean people that you know and who also know you. | 1. How many people do you know who inject drugs or use opioids to get high? 3. How many of those [#1] are 18 years and older? 4. How many of those [#3] live or work in [target area]? 2. How many of those [#4] have you seen in the past one month?  Note: The order of the questions for the network questions was changed on 11/17/2018 (originally asked in numerical order). | Please think about the people living in your area who are at least 15 years of age and who to your knowledge, have injected any type of drug to get high in the past 30 days. How many of these people do you know personally? By this, I mean people that you know and who also know you. |
|  | 6e | $40 for completing the survey and $20 for each eligible peer referral. | $25 for completing the survey, $20 for completing rapid tests for HIV, HCV, and syphilis, and $10 for each eligible peer referral who agreed to participate. | $25 for completing the survey and $10 for each eligible peer referral who agreed to participate. | $20 for completing the survey and rapid tests for HIV, HCV, and syphilis; $10 for each eligible peer referral who completed all study activities (i.e., survey and lab work). | $25 for completing the survey and $10 for each eligible peer-referral (no more than 4 recruits). After a recruit was deemed to be eligible, the recruiter was reimbursed for their time and effort in explaining the study to their peer; a recruited participant did not have to enroll in the study in order for their recruiter to be reimbursed. | $25 for completing the survey and $10 for each eligible peer referral. | $20 for completing the survey and rapid testing, and $10 for each referral who enrolled in the study. |
| *Variables* | 7b | Based on unique code word and coupon number. A Microsoft Access-based coupon manager was used to input coupon information and track recruitment chains. | Based on unique coupon codes. A Microsoft Excel database was used to track recruiter-recruitee pairs. | RDS Coupon Manager | Based on unique coupon IDs. A Microsoft Excel coupon manager spreadsheet was used to track recruiter-recruitee pairs. | Based on unique coupon codes. A Microsoft Access database was used to track recruiter-recruitee pairs. | Based on study ID and coupon number. A tailored Microsoft Excel coupon manager spreadsheet was used to track recruiter-recruit relationships. | Based on coupons. A Microsoft Excel coupon manager spreadsheet and a REDCap database were used to track recruiter-recruitee pairs. |
| *Data sources/ measurement* | 8b | Methods to assess eligibility: A screener survey that asked about date of birth, county, zip code of where the participant last slept, and lifetime use of various drugs (e.g., heroin, fentanyl, cocaine, methamphetamine, pain pills/prescription opioids) to get high or drug injection; where applicable, date of last use was also asked. Additionally, eligibility had to be confirmed by at least 1 of the following: 1. Pill card test (among those who endorsed using an opiate painkiller in the past 30 days); 2. Drug injection questionnaire (among those who endorsed injection of any drug in the past 30 days); and/or 3. Urine toxicology screen. | Methods to assess eligibility: A screener survey that asked about date of birth, county, zip code of where the participant last slept, and lifetime use of various drugs (e.g., heroin, fentanyl, cocaine, methamphetamine, pain pills/prescription opioids) to get high or drug injection; where applicable, date of last use was also asked. | Methods to assess eligibility: A screener survey that asked about date of birth, county, zip code of where the participant last slept, and lifetime use of various drugs (e.g., heroin, fentanyl, cocaine, methamphetamine, pain pills/prescription opioids) to get high or drug injection; where applicable, date of last use was also asked. Additionally, study staff performed a urine toxicology screen to assess recent use (note: although participants were not required to have a positive urine toxicology screen, 91% of the sample tested positive for methamphetamine, an opioid, or both). | Methods to assess eligibility: A screener survey that asked about date of birth, county, zip code of where the participant last slept, and lifetime use of various drugs (e.g., heroin, fentanyl, cocaine, methamphetamine, pain pills/prescription opioids) to get high or drug injection; where applicable, date of last use was also asked. | Methods to assess eligibility: Potential participants were briefly assessed for eligibility when they first contacted study staff (via phone call or text) to find out information about participating. If the potential participant seemed eligible, they were invited to the study site for the screener survey. The screener survey that asked about date of birth, county, zip code of where the participant last slept, and lifetime use of various drugs (e.g., heroin, fentanyl, cocaine, methamphetamine, pain pills/prescription opioids) to get high or drug injection; where applicable, date of last use was also asked. | Methods to assess eligibility: A screener survey that asked about date of birth, county, zip code of where the participant last slept, and lifetime use of various drugs (e.g., heroin, fentanyl, cocaine, methamphetamine, pain pills/prescription opioids) to get high or drug injection; where applicable, date of last use was also asked. | Methods to assess eligibility: Potential participants were briefly assessed for eligibility when they first contacted study staff (via phone call or in-person with a research assistant) to find out information about participating. If the potential participant seemed like they might be eligible, they were invited to the study site for the screener survey. The screener survey asked about date of birth, county, zip code of where the participant last slept, and lifetime use of various drugs (e.g., heroin, fentanyl, cocaine, methamphetamine, pain pills/prescription opioids) to get high or drug injection; where applicable, date of last use was also asked. |
|  |  | Methods to reduce repeat enrollment: A coupon discrepancy checker tool was used to help track the initial seed, any referrals, and if a specific coupon code was redeemed more than once by a participant or assigned to >1 participant. Additionally, cursory checks by study personnel were conducted to help catch any repeat enrollees or discrepancies. | Methods to reduce repeat enrollment: After completing the baseline survey, but before participants were given coupons, they were asked a series of questions to reduce fraud (e.g., maiden name, birth city, eye color, date of birth, first 2 letters of their middle name, other nicknames). Additionally, community-based staff performed a post-hoc review of participant names and characteristics for the purpose of duplicate detection. | Methods to reduce repeat enrollment: Study staff took digital photographs of all participants and recorded detailed descriptions of visible scars and tattoos. Additionally, ~60% of participants also presented a state issued driver’s license or photo ID. Lastly, study sites were located within LHDs, where LHD staff were responsible for all aspects of HIV/HCV/Syphilis testing and notification. Having to provide a name and contact information to 2 entities (study staff and LHD staff) may have discouraged people from providing false information. | Methods to reduce repeat enrollment: Research staff used a secured master spreadsheet to track coupon distribution and reduce repeat enrollment. | Methods to reduce repeat enrollment: Participant details including name, date of birth, hair color, and eye color were recorded in the data manager. These data were used as a reference when a participant came back in to claim a recruitment or confirmatory reimbursement and to reduce repeat enrollment. | Methods to reduce repeat enrollment: When recruits presented for study enrollment, study staff used the coupon manager to verify the status of the recruiter’s coupons. Staff relied on visual recognition and checked recruits’ names against the study’s master enrollment list. | Methods to reduce repeat enrollment: At the beginning of screening, each participant was asked for their first and last name to check in our database to ensure that they were not repeat participants. After enrollment, we cross-referenced paperwork from the state health department to ensure that the participant was not a repeat enrollment. |
| *Bias* | 9 | None | In an attempt to prevent the sample being skewed toward 1 county, KY stopped recruitment in an overrepresented county (see [section 6c](#KY6c) for details). | In an attempt to reduce potential bias related to transportation access, NC increased their recruitment incentive; study staff found that the initial incentive was not enough to cover the cost of gas and the participant's time if they lived far from the static sites, leading to the concern that this could lead to overrepresentation of people who lived in town and close to the field sites. | None | None | In an attempt to reduce potential bias related to transportation access and the large geographic distances in our study areas, the referral coupons included the statement “Let us know if you need a ride” beneath the telephone number of study staff. | None |
| *Study size* | 10 | The target sample size was 100, as this was a pilot/exploratory effort in a research naïve population and a first-time field effort for the research team. The number of participants enrolled (N=174) exceeded the target sample size. | The study sample size was determined based on power calculations for the primary study objectives and with consideration of resource limitations. | The study sample size was determined based on power calculations for the primary study objectives. | The target sample size was 500 participants based on an estimate of the likely recruitment rate. Enrollment remained open at each site for as long as possible until the number of new participants slowed substantially (e.g., 1 new participant in a 7-14 day period). The enrolled participants with outstanding coupons were then advised that the site would be open for 2 more weeks before moving to the next location. | The original planned sample size was 420 based on a power calculation that accounted for a design effect of 4. Due to recruitment challenges, the target sample size was revised to 244 participants. The number of participants enrolled (N=258) exceeded the revised target sample size. | The study sample size was determined based on power calculations for the primary study objectives. The number of participants enrolled (N=174) exceeded the target sample size. Recruitment efforts were stopped when recruitment had slowed substantially, and the study team determined that saturation of potential interested participants had been reached within the pilot geographic outreach areas. | The study sample size was determined based on power calculations for the primary study objectives. |
| **Results** |  |  |  |  |  |  |  |  |
| *Participants* | 13f | Lost coupons: This may have been exacerbated by a percentage of the population being transient. A staff member would go out to meet participants with re-issued coupons, but then this created another challenge when some located their old coupons and were confused. Since study staff often had to look up the referring participant by name (because of lost coupons), they began to rely on REDCap forms to affirm recruitment info and stopped reissuing hard copies. | Trust: During the first year (February 2018 to February 2019), participants were invited to complete a follow-up survey to assess recruitment challenges; the survey was administered to 110 participants. In a question that asked respondents why people were not interested in participating (if they had encountered that in their recruitment attempts), the most common reasons given by their peers were lack of trust that the researchers would keep their information private and that they did not want to talk about private things. This information was used to redesign the coupons to have a greater emphasis on confidentiality, including the Federal Certificate of Confidentiality. | Duplicate recruits: Some concern that participants may try to enroll in both field sites, however there was not any evidence that occurred. The 2 sites were separated by >50 miles on a 2-lane road that ran through a national forest and a river gorge. We believe these geographic barriers limited travel between the 2 field sites. | Duplicate recruits: A few isolated cases where individuals attempted to participate in the study more than once (attempted to enroll at a different study site in a different town); this seemed like a genuine mistake in each circumstance. The same study staff worked at each site, so they were able to quickly identify any attempts at participating more than once. | Trust: Difficulties recruiting the first RDS survey participants. Eligible individuals were initially skeptical of study staff and the idea of a research study, and it was difficult to get participants to appear at the study site. Potential participants who initially called or texted and agreed to participate often failed to show up for their appointment and would not respond to subsequent attempts to reschedule. Study staff gradually addressed this challenge by building trust with participants and encouraging them to use their study coupons to recruit others (and reassure others that the study was not associated with law enforcement, etc.); as word of the study spread, recruitment improved. | Duplicate recruits: 2 attempts at duplicate participation were detected (1 was detected prior to enrollment, the other was discovered after enrollment). The repeat participant's record was removed from the dataset. | Recruitment via seeds: Coupons were often distributed in high trafficked areas such as homeless shelters, and this occasionally caused some confusion as many people would come in for 1 seed, and it was hard to determine who was actually recruited. |
|  |  | Recruitment via seeds: A couple situations where over-enthusiastic recruiters were in communal situations (e.g., homeless shelter, food bank, etc.) and seemed to hand out coupons to whoever would accept them, creating situations where people were let down due to disqualification. This was addressed by having study staff thoroughly explain to recruiters why they could not be compensated for obviously unqualified recruits. Additionally, sometimes multiple enrolled participants would disagree when laying claim to recruits. | Duplicate recruits: Duplication of recruitment did occur; 3 participants evaded detection and successfully enrolled in the study twice. Repeat participants were removed from the dataset. |  | Lost coupons: Participants would sometimes report having lost coupons but still wanting to refer people from their networks to the study. In some cases, staff would replace a set of lost coupons only to have the original coupons return later. As noted in 6c, this contributed to a few participants receiving more than the intended maximum number of coupons per person. |  |  | Duplicate recruits: Duplication of recruitment did occur, but we had the ability to check this based on our data collection methods for HIV/HCV/Syphilis testing. 14 repeat participants and their 18 downstream recruits were identified and subsequently removed from the dataset. |
|  |  |  |  |  | Phlebotomy: Issues with access to phlebotomy in some rural areas which limited options for recruitment sites. This was addressed by partnering with Planned Parenthood of Northern New England who agreed to perform blood draws in several locations. |  |  |  |
|  |  |  |  |  | Accessibility: Limited or non-existent public transportation options meant that recruitment sites needed to be in locations that were most convenient to potential participants. This issue was addressed by identifying sites located as close to town centers as possible while also being located close to a phlebotomy site (e.g., Planned parenthood of Northern New England). Note: Winter weather potentially exacerbated accessibility issues and may have negatively impacted recruitment/enrollment efforts as study staff noted fewer people out and about. We intentionally avoided opening new sites in December/January. |  |  |  |

Abbreviations: ACASI, Audio Computer-Assisted Self-Interviewing; HRS, harm reduction services; IDU, injection drug use; LHD, local health department; PWUD, person who uses drugs; SSP, syringe service program.

^a^ Thresholds determined based on a gender-stratified analysis of preliminary data from the online survey that found that network sizes of 10 and 20 demarcated the top quartile for women and men, respectively. Young AM, Ballard AM, Cooper HLF. Novel recruitment methods for research among young adults in rural areas who use opioids: cookouts, coupons, and community-based staff. *Public Health Rep* 2020; 135(6):746-755.

**Supplemental Table 2.** Non-response/missingness* of key variables overall and by study.

|  |  | **Study** | | | | | | |
| --- | --- | --- | --- | --- | --- | --- | --- | --- |
|  | **Overall** | **IL** | **KY** | **NC** | **NE** | **OH** | **OR** | **WI** |
| **Age** | 0 | 0 | 0 | 0 | 0 | 0 | 0 | 0 |
| **Heroin use**^a^ | 58 (2.0%) | 1 (0.6%) | 0 | 2 (0.6%) | 1 (0.2%) | 0 | 0 | 54 (5.6%) |
| **Fentanyl use**^a^ | 133 (4.6%) | 6 (3.6%) | 28 (8.3%) | 24 (6.9%) | 41 (7.0%) | 12 (4.8%) | 10 (5.8%) | 12 (1.2%) |
| **Methamphetamine use**^a^ | 43 (1.5%) | 0 | 1 (0.3%) | 0 | 20 (3.4%) | 1 (0.4%) | 1 (0.6%) | 30 (3.1%) |
| **HCV antibody status** | 250 (8.8%) | 19 (11.5%) | 1 (0.3%) | 156 (44.6%) | 42 (7.1%) | 17 (6.8%) | 15 (8.6%) | 0 |
| **Homelessness**^b^ | 46 (1.6%) | 2 (1.2%) | 0 | 2 (0.6%) | 2 (0.3%) | 3 (1.2%) | 0 | 38 (3.9%) |
| **Preferred drug for getting high** | 15 (0.5%) | 0 | 0 | 0 | 0 | 0 | 0 | 15 (1.5%) |
| *Includes “Don’t know”, "Refused", and missing responses.  Abbreviations: IL, Illinois; KY, Kentucky; NC, North Carolina; NE, New England (Massachusetts, New Hampshire, Vermont); OH, Ohio; OR, Oregon; WI, Wisconsin.  ^a^ Reference period: past 30 days.  ^b^ Reference period: past 6 months. | | | | | | | | |

**Supplemental Table 3.** Mean degree and degree ratios according to participant characteristics/attributes, by study.

|  | **Study** | | | | | | | | | | | | | |
| --- | --- | --- | --- | --- | --- | --- | --- | --- | --- | --- | --- | --- | --- | --- |
|  | **IL** | | **KY** | | **NC** | | **NE** | | **OH** | | **OR** | | **WI** | |
|  | Mean degree | Degree Ratio | Mean degree | Degree Ratio | Mean degree | Degree Ratio | Mean degree | Degree Ratio | Mean degree | Degree Ratio | Mean degree | Degree Ratio | Mean degree | Degree Ratio |
| **Age** |  |  |  |  |  |  |  |  |  |  |  |  |  |  |
| <25 | 13 | 1.6 | 59 | 1.6 | 72 | 1.6 | 2.8 | 1.2 | 24 | 1.3 | 76 | 2.2 | 24 | 2.4 |
| 25-34 | 10 | 1.3 | 55 | 1.4 | 73 | 1.7 | 2.4 | 1.0 | 42 | 2.2 | 50 | 1.4 | 14 | 1.4 |
| 35-44 | 14 | 1.8 | 47 | 1.2 | 76 | 1.7 | 2.6 | 1.1 | 43 | 2.3 | 44 | 1.3 | 17 | 1.7 |
| 45-54 | 11 | 1.4 | 43 | 1.1 | 72 | 1.6 | 2.1 | 0.9 | 32 | 1.7 | 46 | 1.3 | 9 | 0.9 |
| ≥55 | 8 | Ref | 38 | Ref | 44 | Ref | 2.4 | Ref | 19 | Ref | 35 | Ref | 10 | Ref |
| **Heroin use**^a^ |  |  |  |  |  |  |  |  |  |  |  |  |  |  |
| No | 12 | Ref | 36 | Ref | 44 | Ref | 2.1 | Ref | 24 | Ref | 41 | Ref | 10 | Ref |
| Yes | 11 | 0.9 | 57 | 1.6 | 85 | 1.9 | 2.5 | 1.2 | 41 | 1.7 | 53 | 1.3 | 17 | 1.7 |
| **Fentanyl use**^a^ |  |  |  |  |  |  |  |  |  |  |  |  |  |  |
| No | 11 | Ref | 41 | Ref | 54 | Ref | 2.1 | Ref | 27 | Ref | 49 | Ref | 14 | Ref |
| Yes | 12 | 1.1 | 72 | 1.8 | 93 | 1.7 | 2.6 | 1.2 | 43 | 1.6 | 46 | 0.9 | 21 | 1.5 |
| **Methamphetamine use**^a^ |  |  |  |  |  |  |  |  |  |  |  |  |  |  |
| No | 8 | Ref | 50 | Ref | 42 | Ref | 2.4 | Ref | 29 | Ref | 75 | Ref | 9 | Ref |
| Yes | 12 | 1.5 | 50 | 1.0 | 73 | 1.7 | 2.7 | 1.1 | 40 | 1.4 | 47 | 0.6 | 16 | 1.8 |
| **HCV antibody status** |  |  |  |  |  |  |  |  |  |  |  |  |  |  |
| Negative | 12 | Ref | 43 | Ref | 68 | Ref | 2.3 | Ref | 26 | Ref | 44 | Ref | 13 | Ref |
| Positive | 10 | 0.83 | 55 | 1.3 | 85 | 1.3 | 2.6 | 1.1 | 41 | 1.6 | 55 | 1.3 | 19 | 1.5 |
| **Homelessness**^b^ |  |  |  |  |  |  |  |  |  |  |  |  |  |  |
| No | 10 | Ref | 49 | Ref | 62 | Ref | 2.1 | Ref | 30 | Ref | 36 | Ref | 12 | Ref |
| Yes | 12 | 1.2 | 52 | 1.1 | 82 | 1.3 | 2.7 | 1.3 | 44 | 1.5 | 54 | 1.5 | 18 | 1.5 |
| **Preferred drug for getting high** |  |  |  |  |  |  |  |  |  |  |  |  |  |  |
| Heroin | 9 | 0.9 | 58 | 1.4 | 87 | 1.7 | 2.5 | 1.0 | 39 | 1.1 | 57 | 1.8 | 15 | 1 |
| Methamphetamine | 13 | 1.3 | 54 | 1.3 | 71 | 1.4 | 1.8 | 0.8 | 37 | 1.0 | 44 | 1.4 | 16 | 1.1 |
| Other | 10 | Ref | 41 | Ref | 50 | Ref | 2.4 | Ref | 36 | Ref | 31 | Ref | 15 | Ref |
| Abbreviations: IL, Illinois; KY, Kentucky; NC, North Carolina; NE, New England (Massachusetts, New Hampshire, Vermont); OH, Ohio; OR, Oregon; WI, Wisconsin.  ^a^ Reference period: past 30 days.  ^b^ Reference period: past 6 months. | | | | | | | | | | | | | | |

**Supplemental Table 4.** Mean number of participants recruited and recruitment ratios according to participant characteristics/attributes, by study.

|  | **Study** | | | | | | | | | | | | | |
| --- | --- | --- | --- | --- | --- | --- | --- | --- | --- | --- | --- | --- | --- | --- |
|  | **IL** | | **KY** | | **NC** | | **NE** | | **OH** | | **OR** | | **WI** | |
|  | Mean recruit | Recruit Ratio | Mean recruit | Recruit Ratio | Mean recruit | Recruit Ratio | Mean recruit | Recruit Ratio | Mean recruit | Recruit Ratio | Mean recruit | Recruit Ratio | Mean recruit | Recruit Ratio |
| **Age** |  |  |  |  |  |  |  |  |  |  |  |  |  |  |
| <25 | 1.0 | 2.9 | 0.6 | 0.5 | 0.7 | 0.7 | 0.9 | 1.0 | 0.4 | 0.7 | 0.8 | 1.1 | 0.7 | 1.1 |
| 25-34 | 0.7 | 1.9 | 0.9 | 0.8 | 0.9 | 1.0 | 0.9 | 1.0 | 0.8 | 1.5 | 0.7 | 1.0 | 0.7 | 1.2 |
| 35-44 | 0.7 | 1.9 | 0.9 | 0.8 | 0.9 | 1.0 | 1.1 | 1.2 | 0.9 | 1.6 | 0.9 | 1.2 | 0.7 | 1.2 |
| 45-54 | 0.8 | 2.4 | 0.7 | 0.7 | 0.9 | 1.0 | 0.7 | 0.8 | 0.9 | 1.7 | 0.8 | 1.1 | 0.7 | 1.1 |
| ≥55 | 0.4 | Ref | 1.1 | Ref | 0.9 | Ref | 0.9 | Ref | 0.6 | Ref | 0.7 | Ref | 0.6 | Ref |
| **Heroin use**^a^ |  |  |  |  |  |  |  |  |  |  |  |  |  |  |
| No | 0.5 | Ref | 0.8 | Ref | 0.8 | Ref | 0.8 | Ref | 0.8 | Ref | 0.8 | Ref | 0.6 | Ref |
| Yes | 0.9 | 1.7 | 0.9 | 1.2 | 0.9 | 1.2 | 0.9 | 1.2 | 0.8 | 1.0 | 0.7 | 0.9 | 0.8 | 1.2 |
| **Fentanyl use**^a^ |  |  |  |  |  |  |  |  |  |  |  |  |  |  |
| No | 0.7 | Ref | 0.9 | Ref | 0.9 | Ref | 0.8 | Ref | 0.8 | Ref | 0.9 | Ref | 0.7 | Ref |
| Yes | 0.6 | 0.9 | 0.8 | 1.0 | 0.8 | 1.0 | 1.0 | 1.3 | 0.8 | 1.1 | 0.2 | 0.2 | 0.7 | 1.0 |
| **Methamphetamine use**^a^ |  |  |  |  |  |  |  |  |  |  |  |  |  |  |
| No | 0.5 | Ref | 0.8 | Ref | 0.7 | Ref | 0.9 | Ref | 0.6 | Ref | 0.4 | Ref | 0.6 | Ref |
| Yes | 0.7 | 1.4 | 0.9 | 1. | 0.9 | 1.2 | 1.0 | 1.1 | 0.9 | 1.5 | 0.8 | 1.9 | 0.7 | 1.2 |
| **HCV antibody status** |  |  |  |  |  |  |  |  |  |  |  |  |  |  |
| Negative | 0.6 | Ref | 0.8 | Ref | 0.8 | Ref | 0.8 | Ref | 0.9 | Ref | 0.5 | Ref | 0.7 | Ref |
| Positive | 0.8 | 1.4 | 0.9 | 1.1 | 1.0 | 1.3 | 1.0 | 1.3 | 0.9 | 1.0 | 1.0 | 1.7 | 0.8 | 1.3 |
| **Homelessness**^b^ |  |  |  |  |  |  |  |  |  |  |  |  |  |  |
| No | 0.6 | Ref | 0.8 | Ref | 0.8 | Ref | 0.7 | Ref | 0.8 | Ref | 0.6 | Ref | 0.6 | Ref |
| Yes | 0.8 | 1.2 | 0.9 | 1.1 | 0.9 | 1.1 | 1.1 | 1.5 | 0.8 | 1.0 | 0.8 | 1.4 | 0.8 | 1.4 |
| **Preferred drug for getting high** |  |  |  |  |  |  |  |  |  |  |  |  |  |  |
| Heroin | 1.1 | 3.1 | 0.8 | 0.9 | 1.0 | 1.2 | 1.0 | 1.1 | 0.8 | 1.0 | 0.7 | 1.3 | 0.8 | 1.7 |
| Methamphetamine | 0.8 | 2.2 | 0.9 | 1.0 | 0.8 | 0.9 | 0.5 | 0.6 | 0.9 | 1.2 | 0.8 | 1.4 | 0.7 | 1.5 |
| Other | 0.4 | Ref | 0.9 | Ref | 0.9 | Ref | 0.9 | Ref | 0.8 | Ref | 0.6 | Ref | 0.5 | Ref |
| Abbreviations: IL, Illinois; KY, Kentucky; NC, North Carolina; NE, New England (Massachusetts, New Hampshire, Vermont); OH, Ohio; OR, Oregon; WI, Wisconsin.  ^a^ Reference period: past 30 days.  ^b^ Reference period: past 6 months. | | | | | | | | | | | | | | |

**Supplemental Table 5.** Comparison of unweighted and RDS-weighted prevalence estimates for key variables using tree-based bootstrapping, by study.

|  | **Study** | | | | | | | |
| --- | --- | --- | --- | --- | --- | --- | --- | --- |
|  | **IL** | | | | **KY** | | | |
|  | **Unweighted Sample** | **RDS-I (Age)** | **RDS-I (Drug of Choice)** | **RDS-II** | **Unweighted Sample** | **RDS-I (Age)** | **RDS-I (Drug of Choice)** | **RDS-II** |
| **Heroin use**^a^ | 0.47  (0.32, 0.62) | 0.46  (0.32, 0.61) | 0.46  (0.32, 0.61) | 0.43  (0.28, 0.60) | 0.68  (0.62, 0.71) | 0.67  (0.61, 0.71) | 0.63  (0.55, 0.67) | 0.57  (0.42, 0.66) |
| **Fentanyl use**^a^ | 0.26  (0.17, 0.36) | 0.26  (0.18, 0.37) | 0.26  (0.17, 0.36) | 0.26  (0.14, 0.39) | 0.31  (0.23, 0.39) | 0.30  (0.23, 0.38) | 0.26  (0.18, 0.33) | 0.15  (0.08, 0.22) |
| **Methamphetamine use**^a^ | 0.80  (0.63, 0.90) | 0.78  (0.63, 0.89) | 0.79  (0.63, 0.90) | 0.78  (0.61, 0.89) | 0.79  (0.73, 0.83) | 0.78  (0.73, 0.83) | 0.75  (0.67, 0.80) | 0.62  (0.51, 0.80) |
| **HCV antibody status** | 0.45  (0.34, 0.56) | 0.47  (0.36, 0.58) | 0.44  (0.33, 0.54) | 0.50  (0.34, 0.64) | 0.62  (0.56, 0.69) | 0.60  (0.54, 0.67) | 0.59  (0.54, 0.69) | 0.58  (0.46, 0.78) |
| **Homelessness**^b^ | 0.49  (0.42, 0.57) | 0.47  (0.39, 0.56) | 0.49  (0.40, 0.56) | 0.58  (0.43, 0.71) | 0.36  (0.30, 0.40) | 0.37  (0.31, 0.40) | 0.35  (0.27, 0.39) | 0.36  (0.21, 0.47) |
|  | **NC** | | | | **NE** | | | |
|  | **Unweighted Sample** | **RDS-I (Age)** | **RDS-I (Drug of Choice)** | **RDS-II** | **Unweighted Sample** | **RDS-I (Age)** | **RDS-I (Drug of Choice)** | **RDS-II** |
| **Heroin use**^a^ | 0.66  (0.52, 0.75) | 0.64  (0.50, 0.74) | 0.62  (0.48, 0.72) | 0.48  (0.35, 0.60) | 0.90  (0.86, 0.93) | 0.90  (0.86, 0.93) | 0.90  (0.86, 0.93) | 0.89  (0.84, 0.92) |
| **Fentanyl use**^a^ | 0.49  (0.35, 0.59) | 0.48  (0.33, 0.58) | 0.45  (0.32, 0.55) | 0.36  (0.23, 0.48) | 0.68  (0.63, 0.72) | 0.67  (0.62, 0.72) | 0.67  (0.62, 0.72) | 0.64  (0.58, 0.69) |
| **Methamphetamine use**^a^ | 0.93  (0.90, 0.95) | 0.93  (0.89, 0.95) | 0.92  (0.88, 0.94) | 0.91  (0.84, 0.96) | 0.36  (0.31, 0.41) | 0.36  (0.31, 0.41) | 0.36  (0.31, 0.42) | 0.34  (0.29, 0.40) |
| **HCV antibody status** | 0.65  (0.56, 0.73) | 0.66  (0.57, 0.73) | 0.63  (0.53, 0.71) | 0.71  (0.60, 0.80) | 0.59  (0.52, 0.65) | 0.59  (0.53, 0.65) | 0.59  (0.52, 0.65) | 0.57  (0.50, 0.63) |
| **Homelessness**^b^ | 0.43  (0.39, 0.50) | 0.43  (0.38, 0.49) | 0.43  (0.37, 0.50) | 0.35  (0.27, 0.46) | 0.57  (0.48, 0.63) | 0.56  (0.48, 0.63) | 0.57  (0.47, 0.63) | 0.52  (0.44, 0.58) |
|  | **OH** | | | | **OR** | | | |
|  | **Unweighted Sample** | **RDS-I (Age)** | **RDS-I (Drug of Choice)** | **RDS-II** | **Unweighted Sample** | **RDS-I (Age)** | **RDS-I (Drug of Choice)** | **RDS-II** |
| **Heroin use**^a^ | 0.78 (0.66,0.88) | 0.78  (0.63, 0.88) | 0.77  (0.64, 0.88) | 0.71  (0.57, 0.81) | 0.60  (0.54, 0.71) | 0.58  (0.51, 0.70) | 0.55  (0.48, 0.65) | 0.53  (0.41, 0.68) |
| **Fentanyl use**^a^ | 0.63  (0.50, 0.74) | 0.59  (0.49, 0.69) | 0.62  (0.51, 0.72) | 0.46  (0.32, 0.57) | 0.12  (0.07, 0.19) | 0.13  (0.07, 0.20) | 0.10  (0.06, 0.17) | 0.06  (0.03, 0.11) |
| **Methamphetamine use**^a^ | 0.80  (0.67, 0.85) | 0.79  (0.65, 0.85) | 0.79  (0.67, 0.85) | 0.61  (0.39, 0.73) | 0.97  (0.93, 0.99) | 0.97  (0.93, 0.99) | 0.97  (0.92, 1.00) | 0.98  (0.95, 1.00) |
| **HCV antibody status** | 0.71  (0.66, 0.83) | 0.68  (0.63, 0.78) | 0.71  (0.64, 0.83) | 0.55  (0.51, 0.65) | 0.53  (0.44, 0.60) | 0.53  (0.44, 0.60) | 0.51  (0.43, 0.58) | 0.50  (0.33, 0.65) |
| **Homelessness**^b^ | 0.51  (0.39, 0.57) | 0.50  (0.36, 0.57) | 0.51  (0.39, 0.57) | 0.43  (0.25, 0.54) | 0.68  (0.60, 0.77) | 0.67  (0.59, 0.76) | 0.66  (0.57, 0.76) | 0.55  (0.40, 0.71) |
|  | **WI** | | | |  |  |  |  |
|  | **Unweighted Sample** | **RDS-I (Age)** | **RDS-I (Drug of Choice)** | **RDS-II** |  |  |  |  |
| **Heroin use**^a^ | 0.64  (0.59, 0.69) | 0.63  (0.58, 0.68) | 0.63  (0.58, 0.67) | 0.60  (0.55, 0.66) |  |  |  |  |
| **Fentanyl use**^a^ | 0.19  (0.16, 0.22) | 0.19  (0.16, 0.22) | 0.18  (0.15, 0.21) | 0.16  (0.13, 0.19) |  |  |  |  |
| **Methamphetamine use**^a^ | 0.91  (0.88, 0.93) | 0.91  (0.88, 0.93) | 0.91  (0.89, 0.93) | 0.89  (0.86, 0.92) |  |  |  |  |
| **HCV antibody status** | 0.36  (0.32, 0.39) | 0.36  (0.32, 0.40) | 0.35  (0.31, 0.38) | 0.34  (0.30, 0.39) |  |  |  |  |
| **Homelessness**^b^ | 0.63  (0.59, 0.66) | 0.63  (0.59, 0.68) | 0.63  (0.58, 0.67) | 0.59  (0.54, 0.64) |  |  |  |  |
|  | Data presented as: estimate (95% CI).  Abbreviations: IL, Illinois; KY, Kentucky; NC, North Carolina; NE, New England (Massachusetts, New Hampshire, Vermont); OH, Ohio; OR, Oregon; WI, Wisconsin.  Prevalence estimates calculated using linear regression with tree bootstrapping.  ^a^ Reference period: past 30 days.  ^b^ Reference period: past 6 months. | | | | | | | |

**Supplemental Table 6.** Seed-bias sensitivity analysis - Comparison of unweighted and RDS-weighted prevalence estimates for key variables using tree-based bootstrapping, by study.

|  | **Study** | | | | | | | |
| --- | --- | --- | --- | --- | --- | --- | --- | --- |
|  | **IL** | | | | **KY** | | | |
|  | **Unweighted Sample** | **RDS-I (Age)** | **RDS-I (Drug of Choice)** | **RDS-II** | **Unweighted Sample** | **RDS-I (Age)** | **RDS-I (Drug of Choice)** | **RDS-II** |
| **Heroin use**^a^ | 0.49  (0.31, 0.69) | 0.48  (0.29, 0.66) | 0.47  (0.30, 0.66) | 0.44  (0.22, 0.71) | 0.67  (0.57, 0.71) | 0.67  (0.57, 0.70) | 0.62  (0.51, 0.67) | 0.54  (0.31, 0.63) |
| **Fentanyl use**^a^ | 0.23  (0.13, 0.36) | 0.23  (0.13, 0.37) | 0.23  (0.13, 0.36) | 0.25  (0.12, 0.41) | 0.31  (0.22, 0.39) | 0.30  (0.21, 0.40) | 0.27  (0.18, 0.35) | 0.13  (0.06, 0.22) |
| **Methamphetamine use**^a^ | 0.81  (0.59, 0.95) | 0.79  (0.56, 0.94) | 0.81  (0.60, 0.94) | 0.80  (0.52, 0.95) | 0.79  (0.74, 0.84) | 0.79  (0.73, 0.85) | 0.76  (0.68, 0.81) | 0.64  (0.54, 0.79) |
| **HCV antibody status** | 0.50  (0.38, 0.63) | 0.51  (0.39, 0.64) | 0.49  (0.37, 0.62) | 0.54  (0.33, 0.70) | 0.59  (0.52, 0.65) | 0.58  (0.50, 0.64) | 0.57  (0.50, 0.63) | 0.53  (0.41, 0.72) |
| **Homelessness**^b^ | 0.46  (0.37, 0.55) | 0.44  (0.36, 0.54) | 0.46  (0.37, 0.55) | 0.56  (0.35, 0.72) | 0.36  (0.28, 0.40) | 0.37  (0.28, 0.41) | 0.35  (0.26, 0.38) | 0.39  (0.23, 0.50) |
|  | **NC** | | | | **NE** | | | |
|  | **Unweighted Sample** | **RDS-I (Age)** | **RDS-I (Drug of Choice)** | **RDS-II** | **Unweighted Sample** | **RDS-I (Age)** | **RDS-I (Drug of Choice)** | **RDS-II** |
| **Heroin use**^a^ | 0.68  (0.53, 0.77) | 0.66  (0.49, 0.76) | 0.64  (0.49, 0.73) | 0.46  (0.31, 0.60) | 0.91  (0.88, 0.94) | 0.91  (0.88, 0.94) | 0.91  (0.87, 0.94) | 0.90  (0.87, 0.93) |
| **Fentanyl use**^a^ | 0.52  (0.36, 0.62) | 0.50  (0.34, 0.61) | 0.48  (0.34, 0.58) | 0.38  (0.24, 0.51) | 0.68  (0.62, 0.72) | 0.67  (0.63, 0.72) | 0.68  (0.63, 0.72) | 0.65  (0.60, 0.70) |
| **Methamphetamine use**^a^ | 0.93  (0.89, 0.96) | 0.93  (0.89, 0.95) | 0.92  (0.88, 0.95) | 0.91  (0.83, 0.96) | 0.35  (0.30, 0.41) | 0.35  (0.30, 0.41) | 0.35  (0.30, 0.42) | 0.33  (0.28, 0.39) |
| **HCV antibody status** | 0.67  (0.57, 0.74) | 0.67  (0.57, 0.75) | 0.65  (0.55, 0.72) | 0.71  (0.59, 0.81) | 0.60  (0.53, 0.66) | 0.60  (0.53, 0.65) | 0.60  (0.53, 0.66) | 0.58  (0.51, 0.64) |
| **Homelessness**^b^ | 0.42  (0.37, 0.51) | 0.41  (0.36, 0.49) | 0.42  (0.36, 0.50) | 0.35  (0.26, 0.47) | 0.56  (0.45, 0.63) | 0.56  (0.45, 0.64) | 0.56  (0.45, 0.63) | 0.50  (0.42, 0.57) |
|  | **OH** | | | | **OR** | | | |
|  | **Unweighted Sample** | **RDS-I (Age)** | **RDS-I (Drug of Choice)** | **RDS-II** | **Unweighted Sample** | **RDS-I (Age)** | **RDS-I (Drug of Choice)** | **RDS-II** |
| **Heroin use**^a^ | 0.78  (0.62, 0.91) | 0.78  (0.61, 0.90) | 0.78  (0.60, 0.91) | 0.75  (0.58, 0.92) | 0.58  (0.51, 0.71) | 0.57  (0.49, 0.70) | 0.54  (0.46, 0.66) | 0.47  (0.36, 0.60) |
| **Fentanyl use**^a^ | 0.65  (0.53, 0.81) | 0.61  (0.50, 0.75) | 0.64  (0.51, 0.79) | 0.51  (0.38, 0.61) | 0.10  (0.05, 0.17) | 0.11  (0.05, 0.18) | 0.09  (0.04, 0.14) | 0.06  (0.02, 0.11) |
| **Methamphetamine use**^a^ | 0.83  (0.70, 0.88) | 0.82  (0.70, 0.88) | 0.82  (0.71, 0.88) | 0.66  (0.47, 0.76) | 0.98  (0.96, 1.00) | 0.99  (0.96, 1.00) | 0.98  (0.94, 1.00) | 0.98  (0.96, 1.00) |
| **HCV antibody status** | 0.72  (0.65, 0.87) | 0.68  (0.63, 0.81) | 0.71  (0.64, 0.97) | 0.55  (0.50, 0.79) | 0.51  (0.41, 0.59) | 0.52  (0.41, 0.60) | 0.49  (0.37, 0.57) | 0.58  (0.40, 0.72) |
| **Homelessness**^b^ | 0.54  (0.42, 0.60) | 0.52  (0.36, 0.61) | 0.53  (0.39, 0.60) | 0.48  (0.25, 0.59) | 0.67  (0.57, 0.77) | 0.66  (0.57, 0.77) | 0.64  (0.55, 0.76) | 0.56  (0.41, 0.76) |
|  | **WI** | | | |  |  |  |  |
|  | **Unweighted Sample** | **RDS-I (Age)** | **RDS-I (Drug of Choice)** | **RDS-II** |  |  |  |  |
| **Heroin use**^a^ | 0.65  (0.59, 0.71) | 0.64  (0.57, 0.70) | 0.64  (0.58, 0.70) | 0.60  (0.53, 0.67) |  |  |  |  |
| **Fentanyl use**^a^ | 0.21  (0.17, 0.25) | 0.20  (0.16, 0.24) | 0.20  (0.16, 0.24) | 0.17  (0.12, 0.21) |  |  |  |  |
| **Methamphetamine use**^a^ | 0.92  (0.89, 0.94) | 0.92  (0.89, 0.94) | 0.92  (0.89, 0.94) | 0.90  (0.87, 0.94) |  |  |  |  |
| **HCV antibody status** | 0.36  (0.32, 0.40) | 0.37  (0.33, 0.40) | 0.36  (0.31, 0.39) | 0.35  (0.29, 0.39) |  |  |  |  |
| **Homelessness**^b^ | 0.63  (0.58, 0.68) | 0.63  (0.58, 0.68) | 0.63  (0.58, 0.68) | 0.59  (0.53, 0.65) |  |  |  |  |
|  | Data presented as: estimate (95% CI).  Abbreviations: IL, Illinois; KY, Kentucky; NC, North Carolina; NE, New England (Massachusetts, New Hampshire, Vermont); OH, Ohio; OR, Oregon; WI, Wisconsin.  Prevalence estimates calculated using linear regression with robust confidence intervals.  ^a^ Reference period: past 30 days.  ^b^ Reference period: past 6 months. | | | | | | | |

**Supplemental Table 7.** Seed-bias sensitivity analysis - Unweighted and RDS-weighted measures of association (Relative Risks; Odds Ratios) for the relationship between (a) fentanyl use, (b) heroin use, and (c) age and positive Hepatitis C Virus antibody status without seeds

|  | **(a) Fentanyl use^a^ and**  **HCV antibody status** | | **(b) Heroin use^a^ and**  **HCV antibody status** | | **(c) Age (per 10 years)**^b^ **and**  **HCV antibody status** | |
| --- | --- | --- | --- | --- | --- | --- |
| ***Relative Risk Regression***^1^ | **RR** | **95% CI** | **RR** | **95% CI** | **RR** | **95% CI** |
| **Unweighted** | 1.31 | 1.15, 1.50 | 1.39 | 1.25, 1.56 | 1.02 | 0.97, 1.07 |
| **RDS-I:** Homophily by age | 1.32 | 1.16, 1.50 | 1.42 | 1.26, 1.59 | 1.02 | 0.98, 1.06 |
| **RDS-I:** Homophily by drug of choice | 1.32 | 1.15, 1.52 | 1.39 | 1.24, 1.56 | 1.01 | 0.96, 1.07 |
| **RDS-II:** Individual network size/degree | 1.24 | 0.95, 1.61 | 1.27 | 1.05, 1.54 | 1.03 | 0.98, 1.08 |
| ***Logistic Regression***^2^ | **OR** | **95% CI** | **OR** | **95% CI** | **OR** | **95% CI** |
| **Unweighted** | 1.95 | 1.49, 2.55 | 2.04 | 1.64, 2.52 | 1.05 | 0.93, 1.18 |
| **RDS-I:** Homophily by age | 1.99 | 1.59, 2.49 | 2.08 | 1.66, 2.60 | 1.07 | 0.98, 1.17 |
| **RDS-I:** Homophily by drug of choice | 1.92 | 1.42, 2.59 | 1.99 | 1.61, 2.47 | 1.03 | 0.90, 1.18 |
| **RDS-II:** Individual network size/degree | 1.54 | 0.86, 2.75 | 1.76 | 1.29, 2.41 | 1.10 | 0.98, 1.23 |
| Abbreviations: CI, confidence interval; HCV, hepatitis C virus; OR, odds ratio; RR, relative risk.  ^1^ Relative risks estimated separately in each study using modified Poisson regression and combined across studies using random-effects meta-analyses.  ^2^ Odds ratios estimated separately in each study using logistic regression and combined across studies using random-effects meta-analyses.  ^a^ Reference period: past 30 days.  ^b^ Age modeled as a continuous variable. | | | | | | |
